# A pre-Campanian Ignimbrite techno-cultural shift in the Aurignacian sequence of Grotta di Castelcivita, southern Italy

**DOI:** 10.1038/s41598-024-59896-6

**Published:** 2024-06-04

**Authors:** Armando Falcucci, Simona Arrighi, Vincenzo Spagnolo, Matteo Rossini, Owen Alexander Higgins, Brunella Muttillo, Ivan Martini, Jacopo Crezzini, Francesco Boschin, Annamaria Ronchitelli, Adriana Moroni

**Affiliations:** 1 Department of Geosciences, Prehistory and Archaeological Sciences Research Unit, Eberhard Karls University of Tübingen, Tübingen, Germany; 2 Dipartimento di Beni Culturali, Università di Bologna, Via degli Ariani 1, 48121 Ravenna, Italy; 3 Dipartimento di Scienze Fisiche, della Terra e dell’Ambiente, UR Preistoria e Antropologia, Università di Siena, Via Laterina 8, 53100 Siena, Italy; 4 Dipartimento di Scienze Fisiche, della Terra e dell’Ambiente, Università di Siena, Via Laterina 8, 53100 Siena, Italy

**Keywords:** Prehistoric archaeology, Aurignacian, Human-climate interaction, Lithic technology, 3D, *Homo sapiens*, Open science, Anthropology, Archaeology

## Abstract

The Aurignacian is the first European technocomplex assigned to *Homo sapiens* recognized across a wide geographic extent. Although archaeologists have identified marked chrono-cultural shifts within the Aurignacian mostly by examining the techno-typological variations of stone and osseous tools, unraveling the underlying processes driving these changes remains a significant scientific challenge. Scholars have, for instance, hypothesized that the Campanian Ignimbrite (CI) super-eruption and the climatic deterioration associated with the onset of Heinrich Event 4 had a substantial impact on European foraging groups. The technological shift from the Protoaurignacian to the Early Aurignacian is regarded as an archaeological manifestation of adaptation to changing environments. However, some of the most crucial regions and stratigraphic sequences for testing these scenarios have been overlooked. In this study, we delve into the high-resolution stratigraphic sequence of Grotta di Castelcivita in southern Italy. Here, the Uluzzian is followed by three Aurignacian layers, sealed by the eruptive units of the CI. Employing a comprehensive range of quantitative methods—encompassing attribute analysis, 3D model analysis, and geometric morphometrics—we demonstrate that the key technological feature commonly associated with the Early Aurignacian developed well before the deposition of the CI tephra. Our study provides thus the first direct evidence that the volcanic super-eruption played no role in this cultural process. Furthermore, we show that local paleo-environmental proxies do not correlate with the identified patterns of cultural continuity and discontinuity. Consequently, we propose alternative research paths to explore the role of demography and regional trajectories in the development of the Upper Paleolithic.

## Introduction

Examining the biocultural processes that contributed to the emergence and development of the Upper Paleolithic is a central focus of research in Pleistocene archaeology^1–3^. Until recently, the prevailing scenario suggested that the transition from the Middle to the Upper Paleolithic involved a rapid biological replacement of Neanderthals by *Homo sapiens*. This transition was archaeologically visible thanks to the widespread adoption of bladelet-based lithic technologies assigned to the Aurignacian technocomplex^4^. However, recent reassessments have illuminated the complexity of these biocultural processes, suggesting a more extended period of coexistence and interbreeding among different hominin groups well before the Aurignacian^5–10^. Currently, it is thus plausible to hypothesize that the Aurignacian indicates a second, and possibly more successful, wave of *Homo sapiens* expansion across Europe^11,12^.

**Figure 1:**
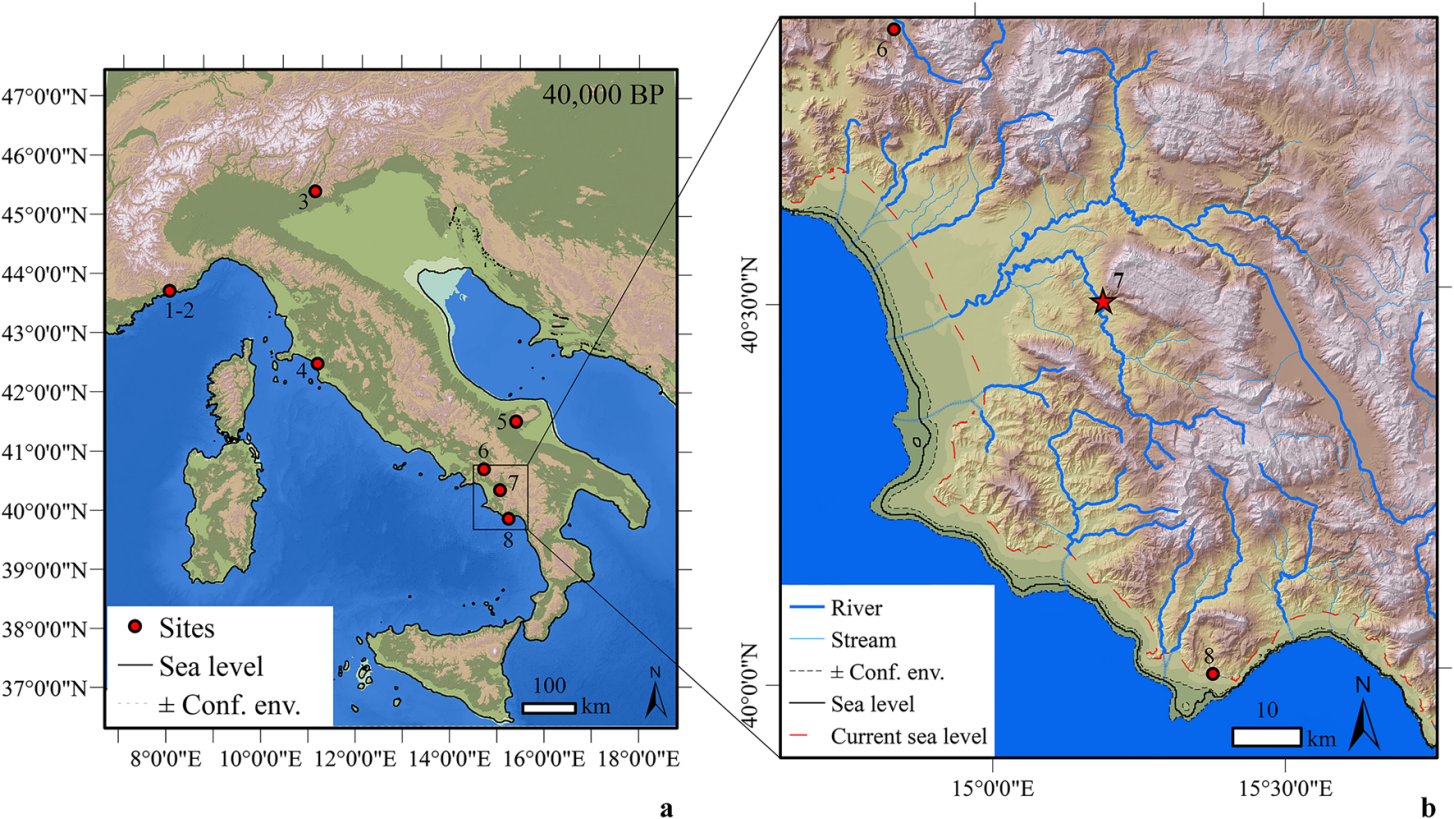
Geographic location of Grotta di Castelcivita and other Aurignacian sites mentioned in the text (**a**), along with a view of the Cilento region showing Castelcivita’s location within its topographical context (**b**). Reported sites: (1–2) Riparo Mochi and Riparo Bombrini, (3) Grotta di Fumane, (4) Grotta La Fabbrica, (5) Grotta Paglicci, (6) Serino, (7) Grotta di Castelcivita, (8) Grotta della Cala. The maps show the paleo-geographic reconstructions of Italy and the Cilento region, taking into account mean sea-level estimations with associated confidence envelopes^20^ at about 40,000 BP (− 62 ± 13 m above the current sea level). The map was generated using ArcGIS^®^10.8: (https://desktop.arcgis.com/en/arcmap/latest/get-started/setup/arcgis-desktop-system-requirements.htm). Source of the Digital Elevation Model: GMES RDA project (https://www.eea.europa.eu/data-and-maps/data/eu-dem#tab-originaldata/eudem_hlsd_3035_europe). Source of the Bathymetry: EMODNET (https://emodnet.ec.europa.eu/en/bathymetry).

Some of the most crucial sites for understanding the early stages of the Aurignacian lie to the south of the Alps and along the Italian Peninsula^13,14^. In northern Italy, the onset of the Aurignacian is dated to ~ 43–42 ka cal BP^15–17^. Notably, at the sites of Riparo Bombrini and Grotta di Fumane (Fig. 1a), two *Homo sapiens* deciduous teeth were discovered within the earliest Aurignacian layers. This discovery provides direct evidence of the makers associated with the lithic assemblages. Moving southward, the Aurignacian begins later. Chrono-stratigraphic evidence suggests that foraging groups persisted in the production of Uluzzian industries in southern Italy, at least until ~ 41 ka cal BP^18,19^.

The earliest expression of the Aurignacian in Italy is recognized as the Protoaurignacian (PA)^21^. In terms of lithic technology, the PA is predominantly characterized by the production of slender and straight bladelets from platform cores through direct, marginal percussion^22^. The reduction procedures linked with this core technology frequently result in the creation of larger blanks, such as blades, for shaping and maintaining core convexities. This process hampers the differentiation between blade and bladelet blanks based on metric and shape attributes^23–25^. Bladelets are frequently modified on one or both edges using marginal retouching^26^, exemplified by sites like Fumane, where retouched bladelets constitute nearly 90% of the tool category^27^.

The development of the Aurignacian in Italy remains a topic of ongoing debate, as only a limited number of stratified sites offer a clear shift from the PA to the so-called Early Aurignacian (EA) cultural variant, unlike some other regions in Europe^28,29^. The EA represents the most investigated cultural variant of the Aurignacian and is considered the initial expression of the Upper Paleolithic in certain regions, such as the Swabian Jura in southwestern Germany^30^. In Table 1, we present a compilation of the most distinctive techno-typological lithic and osseous markers crucial for tracing the chrono-cultural development in the early phases of the Aurignacian. It is important to note that the observed differences are more subtle than previously assumed and should be viewed as tendencies within a generally uniform technological system^22,31^.

**Table 1:** List of the most distinctive features for identifying Protoaurignacian and Early Aurignacian assemblages.

	Protoaurignacian	Early Aurignacian
Lithic technology	- Frequent use of unidirectional platform cores for long and straight bladelets. - Blades frequently obtained from bladelets cores, via initialization or maintenance, as well as through simultaneous reduction procedures. - Occasional presence of carinated cores, both carinated endscrapers and burins.	- Regular use of carinated cores for short, but not twisted, bladelets. - Platform bladelet cores less frequent, with rare narrow-sided/burin-like cores. - Independent production of blades from unidirectional platform cores, with high regional variability due to variations in raw material availability and quality.
Lithic typology	- Bladelets marginally modified, mainly with inverse/alternate retouching. - Burins usually more prevalent than endscrapers.	- Bladelets frequently left unretouched, although alternate or inverse retouching occasionally applied. - Endscrapers as the dominant tool type.
Osseous technology	- Antler points seldom attested and lack modifications at the base.	- Antler points with a split at the basal end of the tool found at certain sites.

Regional markers are excluded from this compilation, which is based on various review papers^22,31,33,36^.

Northern Italy adds complexity to this narrative, as researchers working at Fumane and Bombrini suggest that the PA persisted for a longer duration compared to other European regions^27,32^. Nevertheless, the chronological and archaeological reliability of these findings remains a subject of debate^33–36^. At Riparo Mochi, a PA–EA shift is indeed reported, although updated technological studies are still pending^17^. Other sites associated with the EA are identified in central Italy, particularly in the Circeo region, exemplified by cave sites like Grotta del Fossellone^37,38^.

Given the substantial number of sites and the extensive Paleolithic research tradition^14^, southern Italy emerges as a compelling case study for a more comprehensive understanding of the development of the Aurignacian in southern Europe. One of the most striking features of this region is the presence of volcanoes that have deposited substantial layers of tephra on top and between archaeological sequences throughout prehistory^39,40^. These tephra markers have allowed researchers to establish robust chronological frameworks for the early Upper Paleolithic, despite known challenges associated with obtaining precise radiocarbon dating estimations in the region^41^. The most significant volcanic event within the chronological timespan analyzed in this paper, and indeed the largest in the Mediterranean region, is the Campanian Ignimbrite (CI) super-eruption^42,43^. ^40^Ar/^39^Ar dating places this event at 39.8 ± 0.14 ka^44^. The CI tephra has been identified across several southern Italian localities such as Grotta del Cavallo^19^, Serino^45^, and Grotta di Castelcivita^43,46^. Importantly, this tephra also reached more distant eastern regions, with its identification at sites as far as the Kostenki complex in the Don Region (Russia) and Toplitsa Cave in northern Bulgaria^47–49^, among others^50,51^.

Based on the updated dating, it is suggested that the CI eruption took place slightly after the onset of the Heinrich Event 4 (H4) at 40.2 ka, a period marked by extremely cold and arid conditions^52–54^. This interpretation aligns well with several paleo-environmental and geo-chronological studies that identified the CI within the H4 arid phase^55–59^. Several authors have postulated that the combined action of H4 and CI significantly impacted European ecosystems and human populations^44,48,49,60–63^. Specifically, Giaccio and colleagues^44^ concluded that the synergistic effects of CI and H4 played a pivotal role in the abrupt cessation of both the PA and the Uluzzian across Italy and southeastern Europe.

Utilizing Bayesian chronological data, Banks and colleagues^64^ emphasized a robust correlation between the environmental changes prompted by the onset of H4 and the cultural development of the Aurignacian technocomplex across Europe. The authors suggested that the environmental deterioration was responsible for an expansion of the ecological niche among Aurignacian foraging groups, who underwent rapid cultural adaptations to adjust to the new conditions. According to these authors, the CI eruption would have played no significant role in this process^50^. Archaeologically, this behavioral modification would be evident through the shift from the PA to the EA^28,65^. Likewise, Shao and colleagues^66^ proposed that the harsh and cold climate brought by H4 could have had a profound impact on human groups settled throughout Europe, albeit human habitation remained possible across several regions (see also Paquin and colleagues^67^). It is noteworthy that the scenario proposed by Banks and colleagues encounters challenges from an increasing number of sites exhibiting features consistent with the EA which radiocarbon date to periods before H4, particularly in Central Europe^68,69^. This pattern is also observed in western European sites such as Isturitz^70^ and possibly Lapa do Picareiro^71^. Regardless of the role of H4 in this techno-cultural shift, stratigraphic evidence indicates that when both PA and EA lithic assemblages are recovered from the same site, those displaying more pronounced EA features consistently overlay the PA ones, with no inter-stratifications recorded thus far^50^.

**Figure 2:**
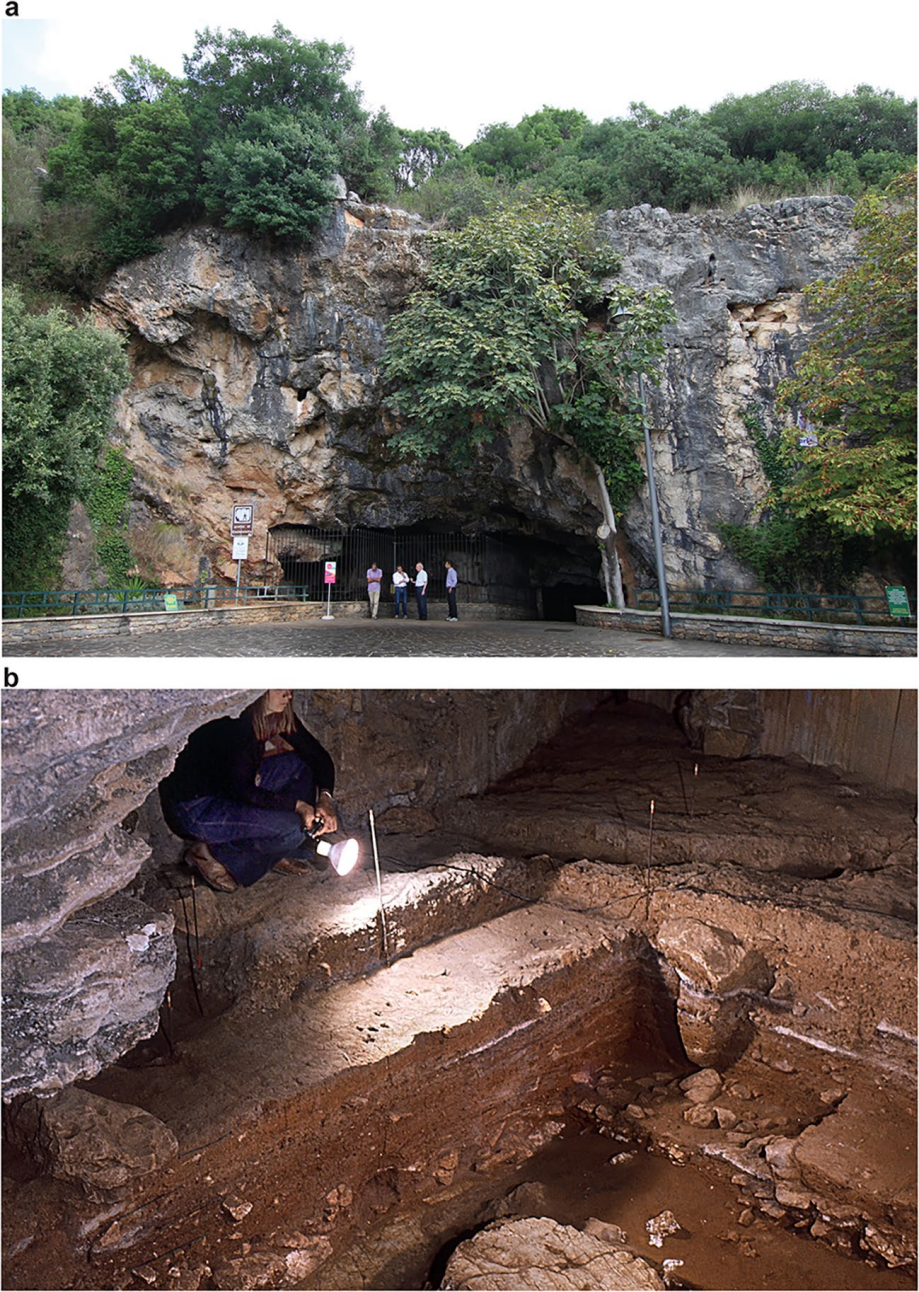
Grotta di Castelcivita, showcasing the cave entrance (**a**) alongside the excavation trench of the Uluzzian and Aurignacian layers (**b**). Photo a credited to A. Ronchitelli, and photo b credited to P. Gambassini. Both photos were edited by S. Ricci.

**Figure 3:**
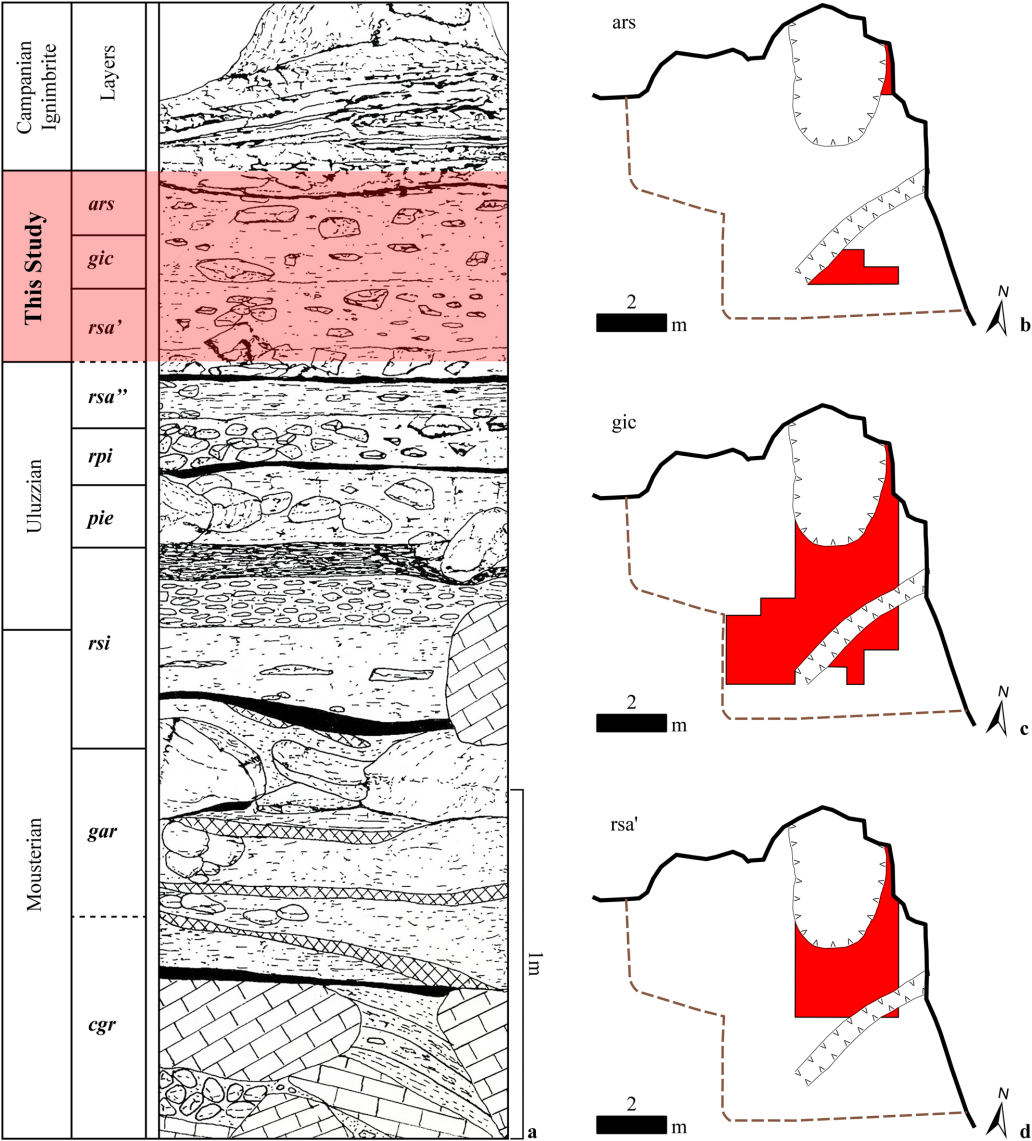
(**a**) Stratigraphic sequence of Grotta di Castelcivita modified after Gambassini^46^. Layers highlighted in red are the ones analyzed in this paper. (**b**, **c**, **d**) Planimetry of the excavation surfaces in *ars*, *gic*, and *rsa’*. The areas colored in red were excavated by P. Gambassini and sampled in this study. For more information about layer *gic*, please refer to Supplementary Fig. S1. Relief and graphics: P. Gambassini, M.P. Fumanal, A. Moroni, and V. Spagnolo.

One of the most significant tephra layers assigned to the CI can be found at Grotta di Castelcivita (Salerno, Campania, southern Italy; Fig. 2). Here, the eruptive units of the CI include Plinian pumice and co-ignimbritic ash layers^43^, and they seal a rich and high-resolution stratigraphic sequence containing evidence of Mousterian, Uluzzian, and Aurignacian industries (Fig. 3 and Supplementary Information). At Castelcivita and throughout Italy, the Uluzzian represents a distinct cultural departure from the preceding Mousterian and stands as one of the most thoroughly studied technocomplexes, owing to its significance in discussions concerning the arrival of *Homo sapiens* in Italy^7^. In terms of behavior, the Uluzzian is characterized by several distinctive features. These include the widespread use of bipolar technique on anvil to produce flakes and bladelets^72–74^, the presence of lunates used in mechanically delivered weapons^75^, the utilization of coloring materials, the production of simple bone tools (e.g., awls), and the use of seashells as personal ornaments. A notable feature is the frequent presence of the tusk shell (i.e., *Antalis* sp.), regarded by some as a hallmark of the Uluzzian^76^

.

At Castelcivita, the Uluzzian is identified in four layers (i.e., upper-*rsi*, *pie*, *rpi*, and *rsa”*). Above *rsa’’*, three layers (*rsa’*, *gic*, and *ars*) were classified as PA based on their position in the stratigraphy just below the CI tephra and the presence of a substantial sample of retouched bladelets^46^. Paolo Gambassini’s study^46^, based on Laplace typology^21^, highlighted the appearance of a miniaturized retouched bladelet type in the layers following the earliest PA, indicating a possible technological change at the site. In the literature, these layers are sometimes referred to as “Protoaurignacian with micro points”^18,74^. Despite this, the technology employed in producing these bladelets has not yet been examined to determine whether technological continuity or discontinuity exists across the sequence. Geological observations by Giaccio and colleagues^43^ suggest that the ca. 50 cm of the *rsa’*–*ars* sequence at Castelcivita accumulated over just a few centuries, commencing at the end of the Uluzzian^18^. Consequently, the site offers an exceptional snapshot of the earliest stages of the Aurignacian before the CI super-eruption.

In this paper, we conduct a comprehensive quantitative investigation of all Aurignacian layers at Castelcivita (*rsa’*, *gic*, and *ars*) and examine the hypothesis that no significant technological changes can be discerned prior to the CI super-eruption. Our approach involves a techno-typological study focused on reconstructing the production methods of laminar blanks, contrasting the results against the defining features of the PA and EA technological systems. Our findings reveal that technological changes at the stratigraphic transition between *rsa’* and *gic* align with a tendency towards the shift to the EA, as already documented in western European sites. Importantly, the process leading to the reliance upon carinated technology at Castelcivita occurred well before the CI super-eruption and the H4, indicating that there is no direct correlation between cold and arid conditions and this technological shift. This study further underscores the distinct regional character of the Castelcivita assemblages, emphasizing the challenges involved in studying Upper Paleolithic population dynamics. Given the regional specificity, we advocate for caution when establishing causal connections between cultural development and environmental fluctuations.

Moreover, from a methodological standpoint, we introduce an approach to lithic analysis that combines traditional technological methods with 3D model analysis, 2D geometric morphometrics, and Open Science practices. Notably, all data manipulation and statistical procedures were carried out within the R programming environment^77,78^ and are made available on Zenodo alongside the datasets required to reproduce our findings^79^. Similarly, 3D models of cores and other noteworthy tools can be accessed in an open-access repository on Zenodo^80^.

## Results

### Overview of the lithic assemblages

The lithic assemblages from layers *rsa’*, *gic*, and *ars *analyzed in this study were recovered during the systematic excavations conducted between 1975 and 1988. All the artifacts were retrieved through the utilization of dry and wet sieving with 1mm mesh screens. Except for the artifacts from the 1988 campaign, all lithics were studied by Gambassini^46^ using the Laplace analytical typology^21^. To thoroughly investigate the variability of blade and bladelet technologies across the sequence, we examined all available lithics without applying any size cut-off. However, we did not sort the rather abundant limestone component, which was predominantly used to produce irregular flakes and is briefly described in Gambassini^46^. We have included only a few previously selected limestone artifacts associated with laminar production (n = 5).

We sorted all cores, complete and fragmented tools, complete blanks, and those fragmented blanks deemed to have played a role in the initialization or maintenance of laminar cores. Table 2 presents a list of the materials analyzed in this study, categorized by lithic class. Layers *gic* and *rsa’* are more abundant in archaeological content, whereas *ars* contains a limited number of artifacts. This is mostly due to the very limited area excavated within this portion of the stratigraphic sequence (see Fig. 3b–d and Supplementary Information). The most predominant raw material in the analyzed layers is the local fine-grained chert, with comparable frequencies (approximately 90%) observed across all layers (Supplementary Information).

**Table 2:** Quantification of the studied assemblages, categorized according to the lithic classes and their layer of provenience.

Layer	Blank	Core	Core-tool	Tool	Total
*ars*	82 (74.5%)	4 (3.6%)	5 (4.5%)	19 (17.3%)	110
*gic*	959 (73.2%)	55 (4.2%)	39 (3.0%)	257 (19.6%)	1310
*rsa’*	1092 (76.5%)	107 (7.5%)	16 (1.1%)	212 (14.9%)	1427
Total	2133 (74.9%)	166 (5.8%)	60 (2.1%)	488 (17.1%)	2847

The noticeable variations in the frequency of lithic categories are attributed to differences in the excavated area for each layer, technological and typological variability across the sequence, as well as the greater number of bipolar cores in *rsa’* (refer to Supplementary Information). The category “core-tool” includes artifacts involved in the production of bladelets (e.g., carinated endscrapers and burin cores) that can also be classified as tools following a typological approach^81^. We have kept these artifacts separate from the core list to facilitate inter-site comparisons. Percentages are provided in brackets.

### Blank production

The most striking feature marking the transition from the Uluzzian to the PA at Castelcivita is the sharp increase in bladelet technologies. Flakes, however, were still produced in all studied assemblages. While not the primary focus of this paper, a description of flake production is included in the Supplementary Information. Notably, the most significant difference identified across the sequence is the decline in the number of bipolar cores in *gic*–*ars* compared to *rsa’* (Supplementary Fig. S2). It is worth noting that bipolar technology is a defining feature of the Uluzzian assemblages at Castelcivita, where it accounts for up to 50% of the lithic production^46,72^. This frequency drops considerably in *rsa’.*

Table 3 presents all cores bearing evidence of blade and bladelet production, which were discarded during the formation of the *rsa’*–*ars* sequence, classified following the criteria outlined by Falcucci and Peresani^82^. The analysis of cores and blanks associated with the initialization and maintenance of core convexities reveals consistent raw material selection strategies and decortication procedures throughout the sequence. In layer *rsa’*, the numerous crested blanks demonstrate that PA knappers frequently utilized core cresting to shape the convexities of bladelet cores (Supplementary Information). Cresting was a commonly employed method to maintain platform cores in the PA, as evidenced by findings from various sites^22,83,84^. The frequency of crested blanks drops in layer *gic* due to the diminished use of platform cores and the increased prevalence of carinated cores (as discussed below). Although crests could be used to initiate blank production on carinated cores, the knapping progression in these cores is known to more effectively auto-maintain the lateral convexities, thus reducing the need for this technical solution^85^.

**Table 3:** Core types associated with the production of blades and bladelets.

Layer	Initial	Burin-like	Carinated	Narrow-sided	Semi-circumferential	Wide-faced flat	Multi-platform	Shatter	Total
*ars*	2 (29%)	1 (14%)	1 (14%)	0 (-)	0 (-)	0 (-)	3 (43%)	0 (-)	7
*gic*	9 (16%)	2 (3%)	29 (50%)	2 (3%)	7 (12%)	2 (3%)	5 (9%)	2 (3%)	58
*rsa’*	8 (15%)	2 (4%)	11 (20%)	2 (4%)	13 (24%)	5 (9%)	10 (19%)	3 (6%)	54
Total	19 (16%)	5 (4%)	41 (34%)	4 (3%)	20 (17%)	7 (6%)	18 (15%)	5 (4%)	119

The classification is based on Falcucci and Peresani^82^, which considers the location and orientation of the flaking surface in relation to the striking platform/s. The table does not include tested cores (n = 2) as the objective of the production could not be assessed. The category of carinated burins is subsumed under the broader carinated category (see the tool list for a breakdown). Rounded percentages are given in brackets.

**Figure 4:**
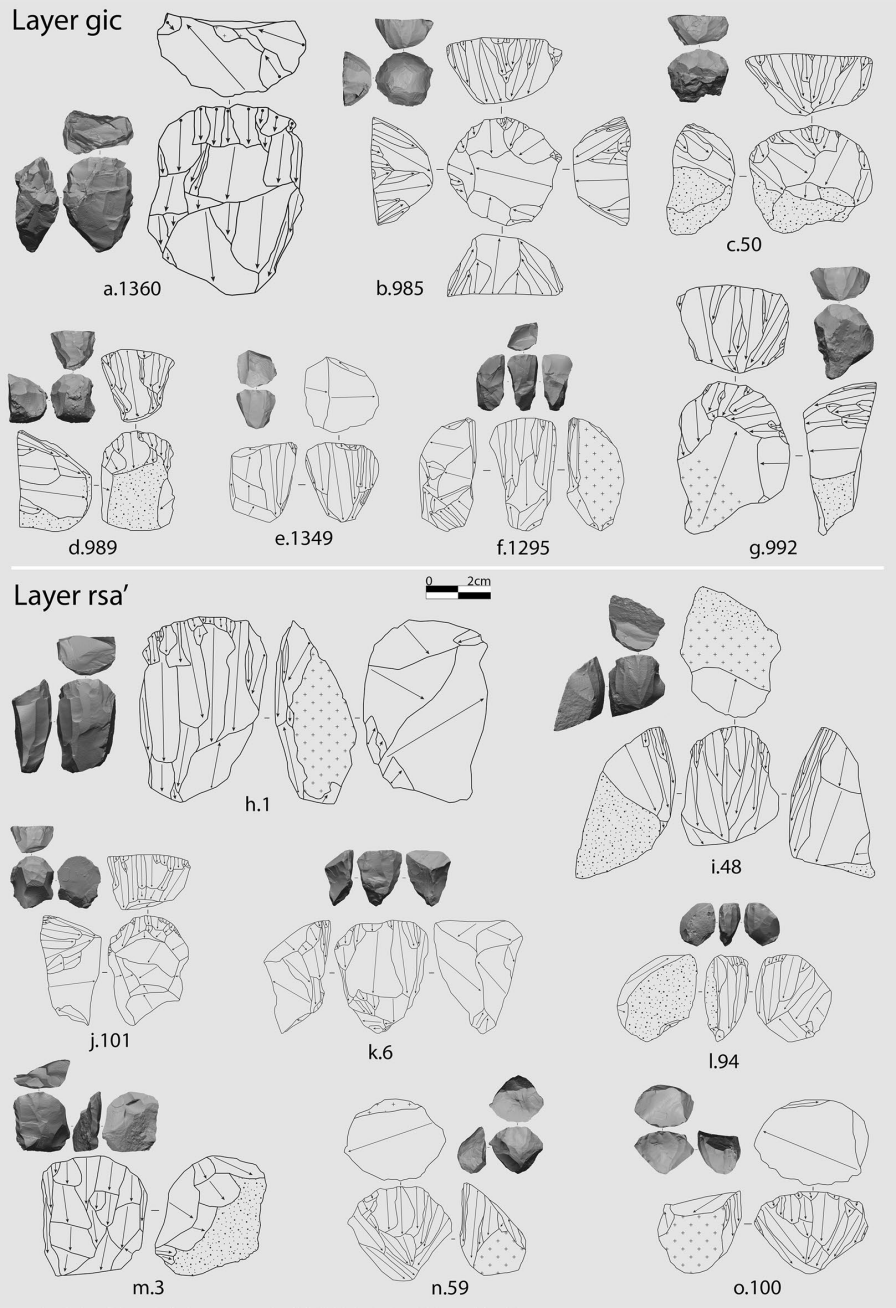
Various perspectives of the 3D models and schematic drawings of cores associated with blade and bladelet production found in layers *rsa’* and *gic*. The numbers shown after the letters are from the dataset created by AF. In both the dataset and the 3D model repository, they are prefixed with “CTC”, which is the common acronym for the site. Cores are classified as semi-circumferential (**a**, **e**, **h**, and **k**), carinated (**b**, **c**, **d**, **g**, **i**, **j**, **l**, **n**, and **o**), wide-faced flat (**m**), and narrow-sided (**f**). The scale refers to the schematic drawing, while the 3D views are half-sized. Drawings by A. Falcucci.

The analysis of crested and fully cortical blanks suggests that blade production was not very common, likely due to constraints related to the size and quality of available raw materials. Exhausted cores provide more insights into the technological variability identified across the sequence (Fig. 4). In *rsa’*, core technology displays a wide range of reduction strategies, with cores oriented according to the longitudinal axis being the most frequent (Fig. 4h–o). Notably, the semi-circumferential strategy is the most common, while narrow-sided and burin cores are less frequent. The high frequency of multi-platform strategies underscores the frequent rotation of cores to fully exploit available raw material volumes.

Intriguingly, carinated technology, the hallmark of the EA, becomes strikingly dominant in *gic* and *ars* (see Fig. 4b–d, and g). When considering all cores with evidence of carinated technology (i.e., carinated cores, carinated core shatters, initial carinated cores, multi-platform cores with at least one carinated reduction sequence; see Supplementary Information), the frequency in *gic* (n = 37; 64%) and *ars* (n = 4; 57%) is in clear contrast to *rsa’* (n = 14; 26%). The technological configuration of carinated cores is however largely consistent across the analyzed layers. Flaking surfaces are primarily wide, striking platforms are plain, and flaking is unidirectional. The significant difference lies in the utilization of shorter flaking surfaces in the upper part of the sequence (Supplementary Fig. S4). Maintenance blanks associated with carinated technology^85,86^ are more prevalent in the upper layers. However, their presence and metric characteristics confirm that carinated technology was also used in *rsa’* (Supplementary Information). Overall, bladelets are the primary production focus throughout the sequence, as shown in Table 4. Analysis of discarded cores and maintenance blanks suggests that independent blade production occurred mainly in *gic*, while simultaneous blade-bladelet production is more prominent in *rsa’* (Supplementary Information).

**Table 4:** Classification of blade and bladelet cores according to the objective of production identified at the time of discard.

Layer	Blade	Blade-bladelet	Bladelet	Total
*ars*	0 (-)	0 (-)	5 (100%)	5
*gic*	3 (6%)	1 (2%)	45 (92%)	49
*rsa'*	0 (-)	4 (9%)	42 (91%)	46
Total	3 (3%)	5 (5%)	92 (92%)	100

Initial cores are not included in this list as scars on initial cores are often related to core shaping operations^22^. Rounded percentages are given in brackets.

**Figure 5:**
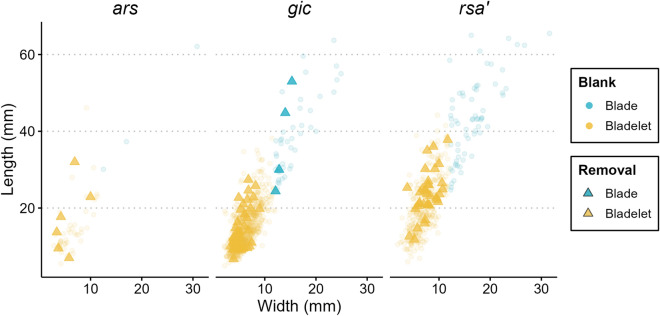
Scatterplots showing the length and width measurements (in mm) of the last successful blade or bladelet removals on laminar cores (triangles) and the dimensions of all complete blades and bladelets (circles) across the studied assemblages. Refer to the legend for corresponding colors.

We measured the last successful blade or bladelet removals on cores and plotted these measurements along with the length and width values of all complete blades and bladelets in the dataset (Fig. 5). In general, the last removals on cores are predominantly classifiable as bladelets, with only a few scars in *gic* (n = 4) attributable to blades. The *gic* scatterplot reveals a notable agreement between the size of the last removals on cores and the complete blades and bladelets. Additionally, there is a noticeable reduction in overlap between the sizes of bladelets and blades at the stratigraphic transition from *rsa’* to *gic*. In *rsa’*, numerous bladelet negatives overlap in length with complete blades, highlighting how the reduction of platform cores resulted in the concurrent production of both blades and bladelets, as commonly observed in the PA^24^. Based on the presented data, layer *gic* demonstrates a substantial increase in the independent production of bladelets, aligning with the increased reliance on carinated core technology (see also Supplementary Information).

**Figure 6:**
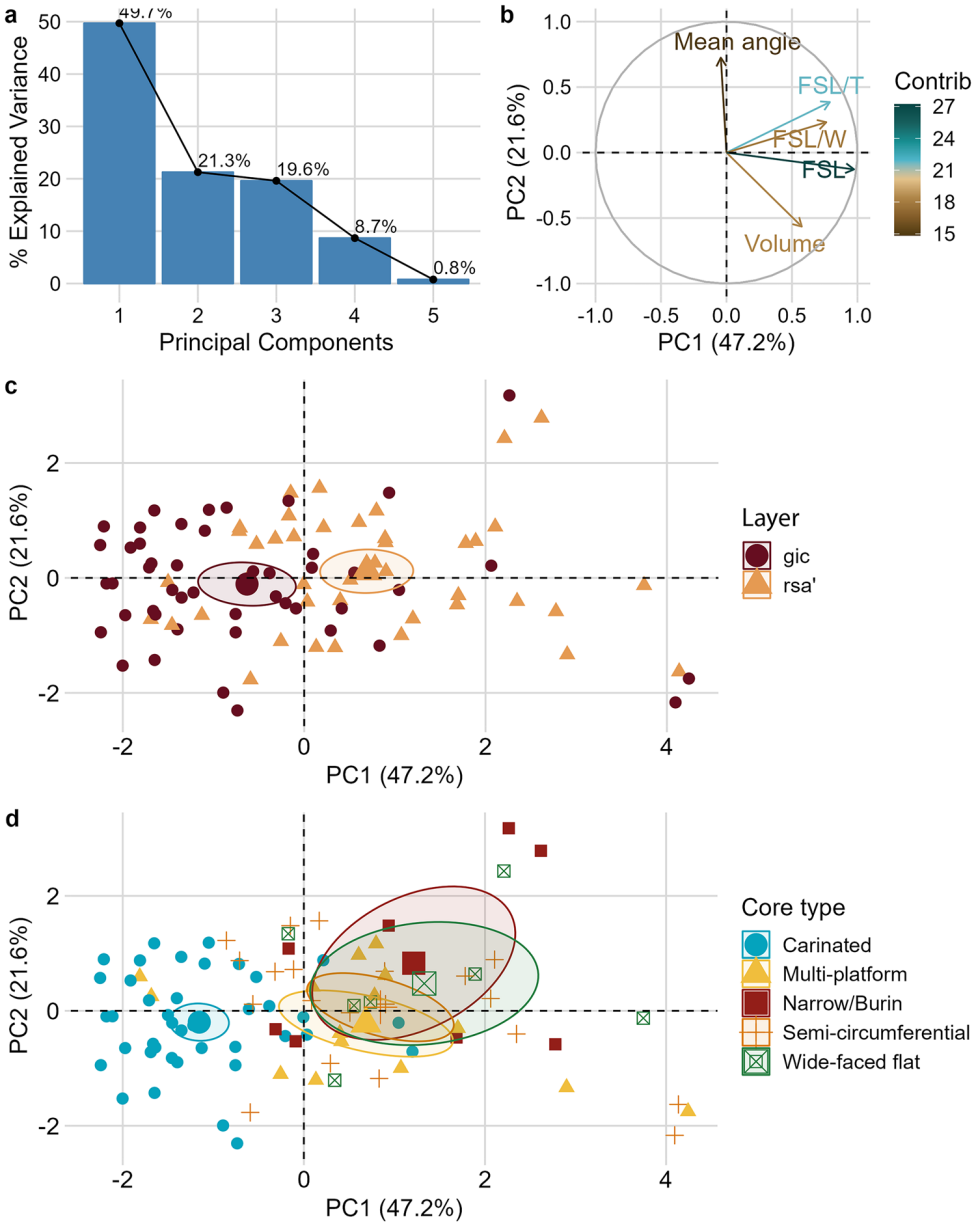
Visualization of the results of the first and second components of the PCA conducted on laminar cores. (**a**) Shows the scree plot and the high percentage of explained variance of the first component. (**b**) Shows a biplot with the contribution of the different quantitative variables to the first and second components. (**c** and **d**) Display the distribution of the studied cores in the PC1 to PC2 space, sorted according to layer (**c**) and core classification (**d**). In (**b**), FSL stands for flaking surface length, FSL/T is the ratio between flaking surface length and core thickness, FSL/W is the ratio between flaking surface length and core width. The category Narrow/Burin includes narrow-sided cores and burin cores, which were merged due to their low number and the similarity in the core geometric configuration. Initial cores and core shatters were excluded from the analysis.

We conducted a Principal Component Analysis (PCA) on five selected quantitative variables measured on blade and bladelet cores to better frame the variability of core types and the technological differences across the sequence. Results are reported in the associated R script and visualized in Fig. 6 and Supplementary Fig. S6. The first three PCs collectively account for approximately 88% of the overall variance, with eigenvalues ranging from 0.99 to 2.4 (Fig. 6a). The graph in Fig. 6b illustrates the strong correlation among shape variables, while the mean striking angle demonstrates an inverse correlation with core volume. The least explanatory variables in the first two principal components are the mean striking angle and the length-to-width ratio of the flaking surface, whereas the length of the flaking surface stands out as the most significant quantitative variable. The qualitative variable “core type” primarily explains the variability observed in the first component, largely distinguishing carinated cores from all other platform cores. Not surprisingly, the variance along the first PC is strongly influenced by the layer of provenience, as *gic* cores are predominantly associated with the carinated strategy (Fig. 6c, d). The significance of these findings lies in their quantitative validation of our core classification, effectively capturing the prominent morphological variability and further emphasizing the clear distinction between gic and rsa’.

The differences identified across the sequence are closely related to the intended production goals. The morphometric analysis of blades and bladelets, detailed in the Supplementary Information, further reinforces the technological shift between *rsa’* and *gic*–*ars*. While most of the compared discrete and metric attributes recorded on blades are quite similar across the sequence, a noticeable reduction in the length of complete blades is observed in *gic*. Conversely to blades, bladelets exhibit more significant variations. Firstly, attributes associated with knapping techniques suggest the use of more marginal percussion in *gic*. In profile view, bladelets from *gic* are straighter and less frequently twisted. The knapping progression in *gic* is often unidirectional convergent. Finally, bladelets recovered in *gic* and *ars* are smaller in terms of length, width, and thickness.

### Tool analysis

The classification of tool types reveals significant variations (Table 5 & Supplementary Figs. S13–S15). Retouched bladelets are consistently the most numerous tool type throughout the stratigraphic sequence, with the highest frequency in *gic*. Notably, the number of endscrapers, many of which can be further classified as carinated and associated with bladelet production, clearly increases in *gic*–*ars*. Nosed endscrapers, either flat- or thick-nosed (Supplementary Fig. S14b, d) are rare. Burins are consistently less frequent than endscrapers, with the sole exception being in *ars*; however, its small sample size requires caution. In some cases, burins were classified as bladelet cores, being either carinated (e.g., Supplementary Fig. S15) or displaying multiple bladelet negatives. The latter exhibit a flaking surface oriented along the narrow, longitudinal side of the blank (e.g., Supplementary Fig. S13a). From a typological perspective, the two carinated burins from *rsa’* can be further classified as busked (Supplementary Fig. S13b) and *Vachons* (Fig. 4l) burins due to the presence of a distal notch in the former, and the extension of the bladelet negatives along the ventral face in the latter. These burins display the detachment of rather short and curved bladelets. Several flakes and blades with lateral retouch were also identified, especially in the lowermost layer. Blades are modified with direct retouch, with the exception of one blade with inverse retouch from *rsa’*. In this layer, three blades display direct unilateral stepped retouch (e.g., Supplementary Fig. S13f), reminiscent of the so-called Aurignacian retouch^87^. The Aurignacian retouch is also a characteristic feature of a few flakes at Castelcivita. On the other hand, several flakes display very slight and localized edge modifications. It is worth noting that we did not include many of the so-called denticulated flakes that are listed in Gambassini^46^. Many of the edge traces on these flakes are discontinuous, irregular, and alternating, which likely reflect the presence of taphonomic and trampling scars^88,89^. Overall, when tools are sorted according to blank type flakes are the second most frequently used blanks, following bladelets (Table S30). Regarding blank technology, tools are primarily made from blanks produced during the optimal production phase (Table S31).

**Table 5:** General overview of the main tool categories recovered across the studied sequence with percentages provided in brackets.

Typological classification	*ars*	*gic*	*rsa’*
Burin simple	3 (12.5%)	2 (0.7%)	8 (3.5%)
Burin carinated	1 (4.2%)	2 (0.7%)	2 (0.9%)
Burin multiple	1 (4.2%)	2 (0.7%)	2 (0.9%)
Composite tool	1 (4.2%)	6 (2.0%)	1 (0.4%)
Endscraper plain	0 (-)	30 (10.1%)	11 (4.8%)
Endscraper carinated	2 (8.3%)	25 (8.4%)	7 (3.1%)
Endscraper flat-nosed	1 (4.2%)	1 (0.3%)	0 (-)
Endscraper thick-nosed	0 (-)	2 (0.7%)	1 (0.4%)
Rabot	1 (4.2%)	6 (2.0%)	4 (1.8%)
Retouched blade	0 (-)	11 (3.7%)	23 (10.1%)
Retouched bladelet	12 (50.0%)	191 (64.5%)	125 (54.8%)
Retouched flake	2 (8.3%)	9 (3.0%)	25 (11.0%)
Scaled piece	0 (-)	2 (0.7%)	8 (3.5%)
Truncation	0 (-)	6 (2.0%)	8 (3.5%)
Undet. retouched piece	0 (-)	1 (0.3%)	3 (1.3%)
Total	24	296	228

*Undet* Undetermined.

#### Retouched bladelets

**Figure 7:**
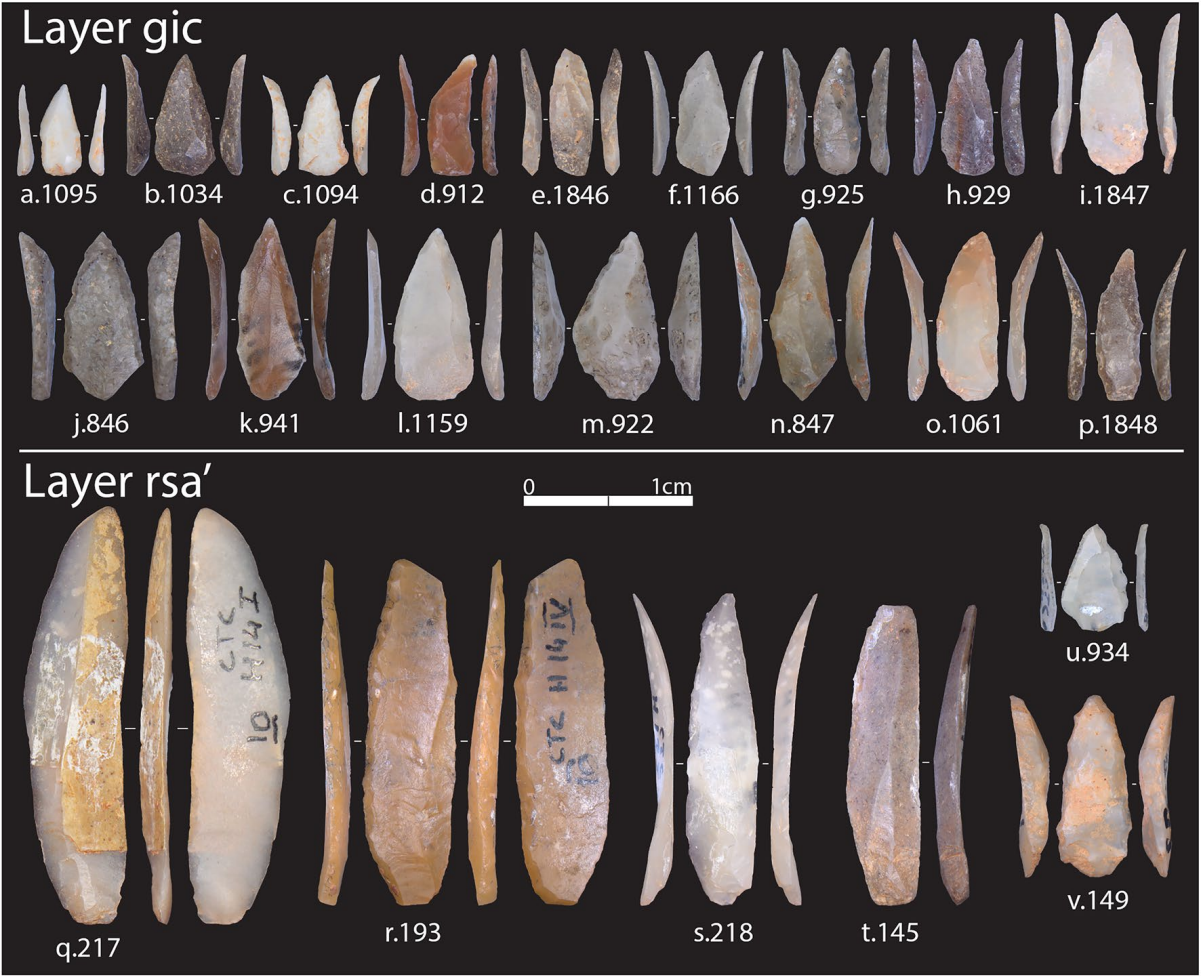
Selection of retouched bladelets from *rsa’* and *gic*. Additional photos of retouched bladelets from *gic* and *ars* can be found in Supplementary Figs. S14–S15. The number following the alphabetical list corresponds to the ID assigned by AF during the techno-typological analysis (refer to the provided dataset for details). Tools are sorted by the position of the retouch: direct bilateral retouch (**a**, **c**–**f**, **h**–**p**, **s**, and **v**), direct unilateral (**b**, **g**, and **u**), inverse (**q**), and alternate (**r** and **t**). Photos by A. Falcucci.

**Figure 8:**
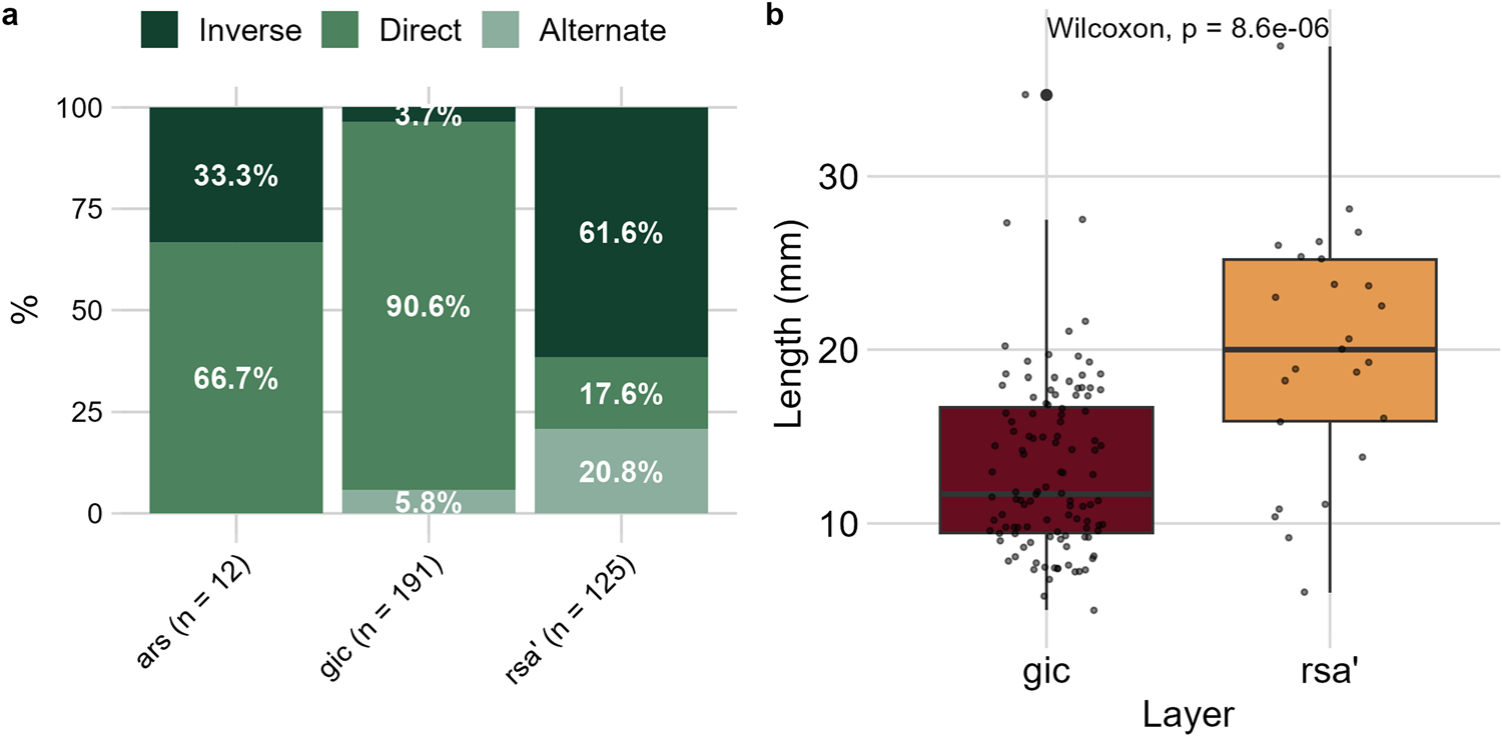
(**a**) Displays the percentage of bladelets modified by alternate, inverse, and direct retouching. (**b**) Shows boxplots with jittered points of length values (in millimeters) for all complete retouched bladelets, along with the results of the Wilcoxon test, confirming significant differences between the two assemblages. Width and thickness values, along with metric data tables, are available in the Supplementary Information.

A total of 328 retouched bladelets were recovered from the studied layers, with the highest number found in *gic* (Supplementary Information). Castelcivita has provided an exceptional number of complete retouched bladelets (Fig. 7 and Supplementary Figs. S14–S15), especially in layer *gic*, representing one of the highest proportions in the Aurignacian^26^. A notable difference between layers is the choice of retouching techniques (Fig. 8a and Supplementary Table S35). In layer *rsa’*, inverse retouch is the predominant technique, followed by alternate and direct retouching in nearly equal proportions. This pattern shifts significantly in *gic*, where bladelets are almost exclusively modified using direct retouch. In *gic*, the direct retouch affects more often both edges of the bladelets (Supplementary Table S36). Many of these artifacts were classified as micro-points by Gambassini^46^, who correctly emphasized their uniqueness in the context of the Italian and, more broadly, European Aurignacian. In terms of metrics, retouched bladelets from *gic* are smaller in all linear dimensions (Fig. 8b and Supplementary Fig. S16), although variation within this pattern exists (see below).

### 2DGM analysis

**Figure 9:**
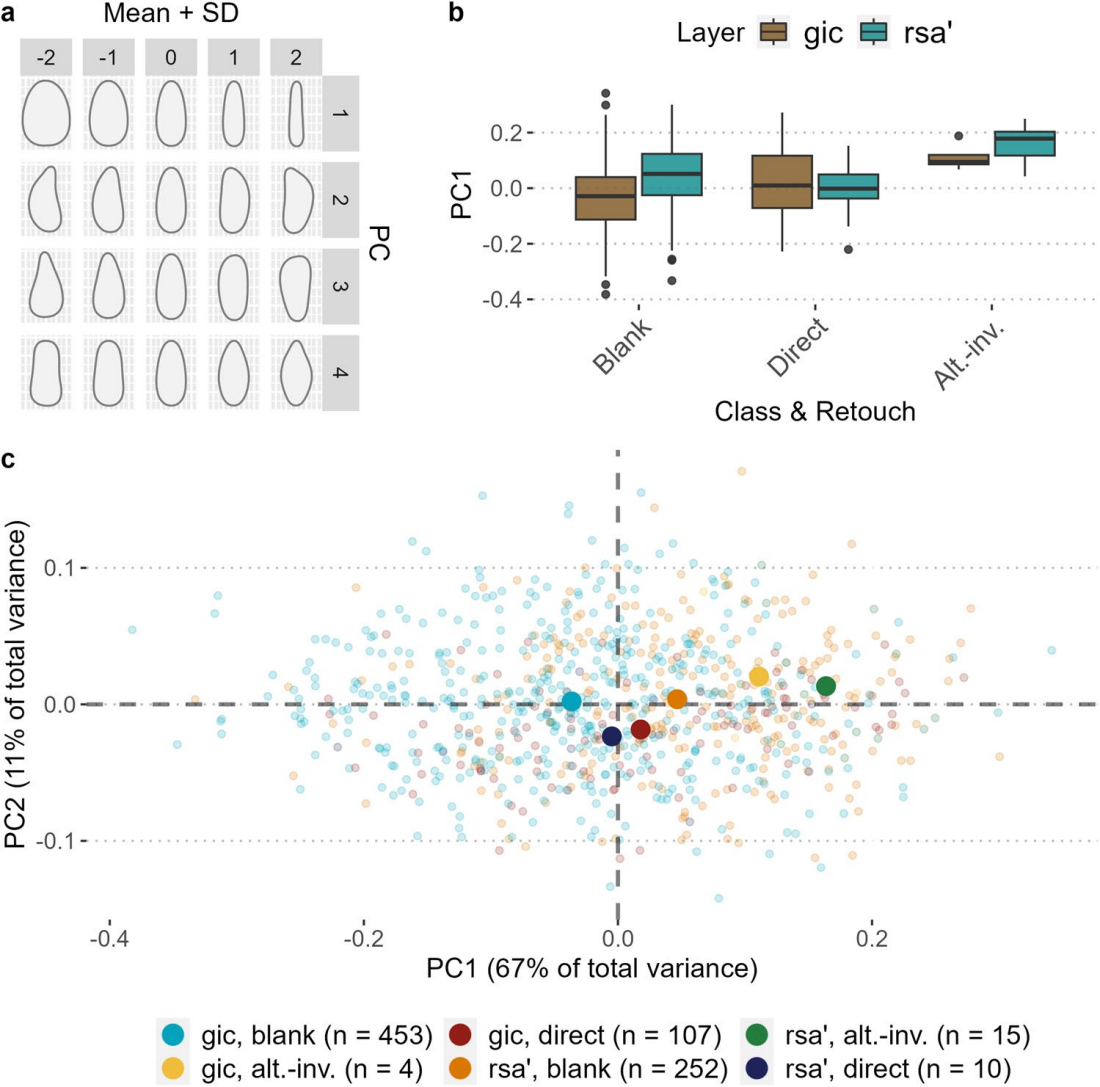
Results of the 2D shape analysis of the complete bladelet dataset. (**a**) Displays the shape variation across the first four PCs (SD stands for standard deviation). (**b**) Shows the boxplots comparing the PC1 scores of the analyzed datasets from *gic* and *rsa’*, sorted according to the presence and type of retouch. (**c**) Is the scatterplot of PC axes 1 and 2 with the mean values of each group displayed as a larger dot. Alternate and inverse retouching are merged into a single category labeled as “alt.-inv.”.

To further explore outline shape variability among bladelets and its relationship with size, we conducted Elliptic Fourier Analysis (EFA) on the 2D outlines of all complete bladelets, both retouched and unretouched (n = 841). The PCA on the Elliptic Fourier descriptors reveals significant variance across the study sample. The first four PCs account for 90% of the total variance (Supplementary Fig. S17). PC1 mainly describes elongation, with blanks on the negative axis being stouter. A Spearman correlation test indicates that PC1 is primarily influenced by variations in the length of the blanks (r^2^ = 0.56, *p* < 0.01; Supplementary Fig. S18). PC2 captures distal symmetry, while PC3 and PC4 relate to the convergence or divergence of the base and apex (Fig. 9a). The PERMANOVA test rejects the hypothesis that the means of all groups are equal (F = 19.836, *p* < 0.01).

The Pairwise Euclidean distances and the mean shapes of artifacts categorized by class (i.e., tool or blank) and retouch position provide additional information on the identified variability (Supplementary Fig. S19). First, non-modified bladelets from *gic* are statistically different from the same group in *rsa’*. Second, the main expectation of the comparisons with the tools is that retouched bladelets are more elongated due to edge modifications affecting the length-to-width ratio. This pattern was well demonstrated through a 3DGM analysis on a sample of PA bladelets from northern Italy^90^. Overall, this expectation is met across all mean shape comparisons, with the exception of the comparison between non-modified bladelets and bladelets with direct retouch from *rsa’*. The PCA biplot in Fig. 9c and the PC1 boxplots in Fig. 9b also confirm this discrepancy as the bladelets with direct retouch plot more towards the negative axis of PC1, instead of the opposite, as in all other cases.

## Discussion

### Contextualizing the lithic assemblages from layers *rsa’*, *gic*, and *ars*

Castelcivita contains one of the most critical sequences for tracking the shift towards the EA cultural variant in southern Italy. The sedimentary complex includes three Aurignacian layers that accumulated within a relatively short timeframe, estimated to be in the order of a few hundred years, just before being sealed by the CI tephra. This eruptive event provides us with an exceptionally well-dated and stratigraphically robust snapshot of the ongoing cultural processes.

In this study, we tested the hypothesis that the PA was a relatively stable technological system prior to the CI super-eruption^44^ by employing a varied analytical approach. Significantly, we identified a notable techno-cultural shift at the stratigraphic transition between layers *rsa’* and *gic*, prompting us to reject the initial working hypothesis. From a lithic technological standpoint, the *gic* assemblage is characterized by the use of carinated technology to produce miniaturized bladelets, which aligns well with the most remarkable trait of the EA, as extensively documented across western Europe^28,30, 33, 36^. While the CI super-eruption might have led to additional modifications in the subsistence systems of foragers settled in the region, this finding emphasizes that the shift was not influenced by this geological event. Furthermore, it compellingly demonstrates that technological changes occurred within a relatively short timeframe across a broad geographic area, encompassing at least western and southern Europe.

The lithic assemblage retrieved from the lowermost layer *rsa’* is largely comparable with other PA assemblages excavated throughout Italy. The techno-typological similarities are particularly evident in the frequent production of bladelets with sub-parallel edges from platform cores (i.e., cores oriented along the longitudinal axis of the raw material blank) and a preference for the use of inverse retouching. In particular, these characteristics have been well-documented at PA sites along the Tyrrhenian coast of Italy, including Grotta La Fabbrica^91^, Mochi^92^, and Bombrini^93^. While there are evident similarities, techno-typological connections are less pronounced with the early PA assemblages from Fumane in northeastern Italy^94^. Notably, the lowermost PA stratigraphic unit at Fumane (i.e., A2) site exhibits a more distinct focus on isolating narrow and convergent flaking surfaces to produce pointed bladelets^22,82^, which were modified through a varied range of retouching techniques^26,90^. The observed similarities among Tyrrhenian sites, in comparison to Adriatic Italy, may suggest that foragers settled in this area and/or their ideas traversed more frequently the north-to-south axis along the Tyrrhenian coastline. This geographic patterning within Italy is worthy of future examination, as it seems to align with data available from other Upper Paleolithic periods, such as the middle Gravettian^13,14, 95,96^.

The available chronological framework supports a north-to-south terrestrial diffusion route of the PA^97^. Notably, the oldest PA occupations at Mochi on the Ligurian coast coincide with the modeled age for the onset of the Uluzzian at Castelcivita^16,18^. The Uluzzian is currently attributed to *Homo sapiens*^7,19^, and this fragmented cultural landscape suggests that complex biocultural processes were ongoing between at least 44 and 40 ka. Although this study did not directly address the transition from the Uluzzian to the PA, we are inclined to describe the beginning of the PA at Castelcivita as a rather abrupt event. The sharp increase in bladelet production, primarily achieved through direct freehand knapping, contrasts sharply with the frequent production of flakes and bladelets from bipolar cores in the preceding Uluzzian layers^72,73^. However, it is worth noting that a few carinated cores and retouched bladelets have also been described in these layers^46^. On the other hand, the occurrence of bipolar cores in *rsa’*, which steadily decreases towards the upper layers of the sequence, does not constitute solid evidence for discussing cultural continuity. Geologically, *rsa’’* and *rsa’* could not be separated during fieldwork due to their sedimentological similarity and the presence of thin sterile layers only in some areas of the excavation. Moreover, bipolar technology is a common feature throughout the European Upper Paleolithic^98^, particularly in southern Italy^14^. Future research will be designed to investigate this aspect more comprehensively by comparing the *rsa’’* and *rsa’* assemblages through a quantitative and multi-disciplinary approach.

### On the absence of correlation between environmental proxies and techno-cultural changes

The most notable finding of this study is the identification of a rapid techno-cultural change occurring at the stratigraphic transition between *rsa’* and *gic*. However, instead of being an abrupt technological shift with no links with the preceding layer *rsa’*, the results of our technological study suggest that the defining features of the *gic* assemblage developed within a clear PA technological background, probably as a result of a combination of cultural transmission as well as innovation processes^99^. When quantitatively comparing *rsa’* and *gic*–*ars*, we found technological similarities across different hierarchical categories^100^ of lithic production referring to all phases of the core reduction sequence. In this framework, we argue that the use of carinated technology, beginning already in *rsa’* and becoming predominant in *gic*, is particularly relevant. The process towards a more independent production of bladelets within the Aurignacian technocomplex is thus to be intended as progressive and bearing a chronological component. For instance, the earliest PA assemblages in northern Italy seldom report the presence of carinated cores^22,93^, unlike the *rsa’* assemblage at Castelcivita. In western Europe, the transition from the PA to the EA is also not abrupt. Particularly, the presence of carinated technology steadily increases starting from the earliest PA assemblages at sites such as Isturitz, La Viña, Labeko Koba, and Les Cottés^84,101–103^.

Overall, our study shows that the cultural processes leading to the EA in southern Italy were already in motion before the CI super-eruption and were not influenced by the alternation of cold and temperate cycles. This observation shows how finding a direct causality between environmental variations and cultural change is extremely challenging when looking at a single site. Considering the sedimentological, anthracological, and archaeozoological evidence available at Castelcivita, we find that cold and arid conditions are more closely linked to *rsa’*, which contradicts the view of the PA as a technocomplex adapted to warmer conditions^64^. Although further studies are needed, we are inclined towards associating the late Uluzzian and the PA with the short cold stadial GS 9/10 identified in the NGRIP2 oxygen isotope curve^104–106^. In this context, *gic* would be linked to the interstadial GI9, while *ars*, where a new cold phase was detected (see Supplementary Information), to the onset of H4. Notably, the most pronounced cultural shift at the site coincides with the formation of layer *gic*, marked by a significant presence of arboreal vegetation around the cave. The weak correlation of climatic events to cultural change is further illustrated by the absence of a marked environmental shift during the transition from the late Uluzzian to the PA, as well as by the clear climatic shift identified between layers *gic* and *ars*, which are in turn archaeologically indistinguishable. Additionally, it is noteworthy that the impact of H4 in Italy may have been less substantial compared to European regions north of the Alps^107^. For instance, the analysis of a stalagmite from Apulia suggests that southern Italy did not undergo dramatic climate oscillations with the onset of H4^108^. This contrasts with southwestern France, where H4 had a major impact on the environment, establishing a cold steppe dominated by reindeer^109^.

### Exploring alternative scenarios and mapping future research trajectories

The distinctive features observed in the *gic*–*ars* layers at Castelcivita have opened avenues for future scientific exploration. Remarkably, the pronounced regional signal characterizing these assemblages sets them apart from other sites across Europe assigned to the EA. Castelcivita stands out as the sole site where carinated technology is associated with the frequent modification of bladelets through direct marginal retouching, a virtually unknown trait in the EA from both Central and Western Europe^28,30,36^. Although retouched bladelets are commonly associated with the PA, it is crucial to highlight that the tools in *gic*–*ars* are rarely assignable to the larger and straighter Dufour subtype Dufour bladelets^81^, which are typically modified through alternate or inverse retouching^26,81^. Notably, bladelets with marginal retouching remain a consistent feature of the Italian Aurignacian throughout its entire temporal range. Prominent examples include the sequences of Fumane and Paglicci, which extend to approximately 36 ka cal BP^15,27^ and 33 ka cal BP^43,110^, respectively.

The miniaturized retouched bladelets from layer *gic* stand out as unique within the Aurignacian context, as noted in the previous typological study^46^. The 2DGM assessment confirmed that their shape is influenced by the utilization of carinated technology, resulting in the production of less elongated bladelets (see PC1 in Fig. 9) compared to the slender bladelets resulting from the platform core technology characteristic of the PA^86^. The function or functions of these bladelets remain unclear, prompting planned functional studies to evaluate whether they were hafted into multi-component hunting tools. In layer *rsa’*, only a few miniaturized bladelets with direct retouch were identified. Several factors, including the absence of a sedimentary hiatus between the two layers, the complex formation processes typical of cave sites^111,112^, and the challenges in establishing a straightforward correlation between field layers and archaeological diachronic changes^113^ may have contributed to this outcome. However, it is crucial to note that the occurrence of post-depositional events at the site is limited. The overall integrity of the studied assemblages is robustly supported by the marked variations observed in lithic, faunal, and sedimentological contents.

Differences between the *gic*–*ars* layers and other EA assemblages across Europe extend beyond lithic artifacts to include osseous artifacts and personal ornaments. At Castelcivita, there is limited evidence for the use of bone tools in the examined layers. Only an awl made from a roe deer metapodial is described from the excavations led by Gambassini of layer *gic*^46^. Conversely, no bone tools are yet described from layers *rsa’* and *ars*^76^. Split-based antler points^114,115^ have not been recovered at Castelcivita. Available data suggests that the split-based points recovered across Italy are generally dated after the CI super-eruption^116^, indicating that this tool type spread south of the Alps in a subsequent phase of the EA^27^.

**Figure 10:**
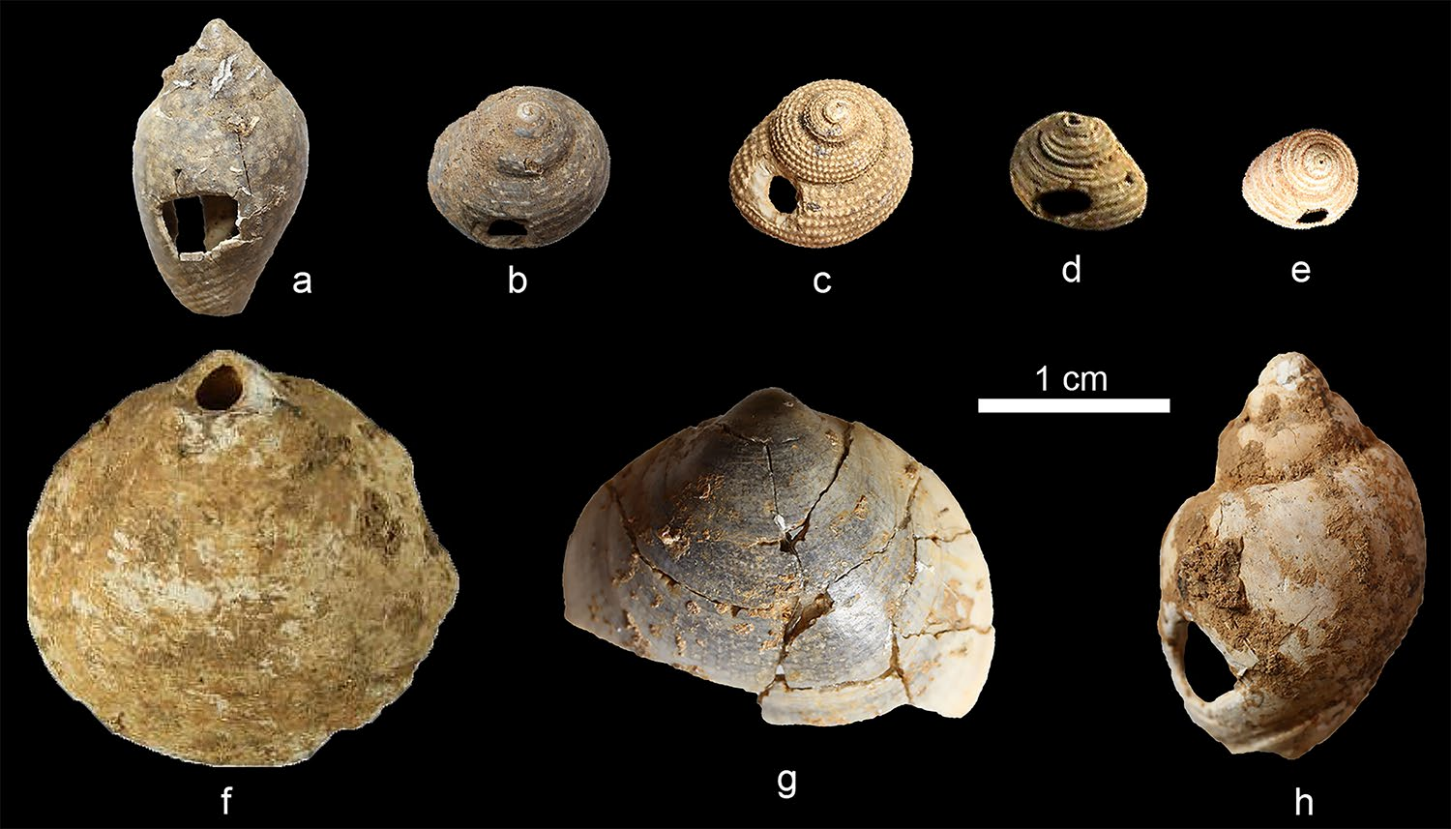
Seashells used as personal ornaments recovered in layer *gic*: *Columbella rustica* (**a**), *Clanculus jussieui* (**b**), *Clanculus corallinus* (**c**), *Homalopoma sanguineum* (**d**, **e**), *Glycymeris nummaria* (**f**, **g**), and *Tritia mutabilis* (**h**). Photos: V. Spagnolo and S. Ricci, edited by A. Falcucci.

Unlike other Aurignacian sites in northern Italy and beyond^76,117^, foragers visiting Castelcivita exclusively utilized seashells as personal ornaments (Fig. 10), with no evidence of other raw materials such as teeth, steatite, or bone being used. The most common seashell species is *Homalopoma sanguineum*, followed by *Glycymeris* sp. and *Pecten jacobaeus*^118^. The majority of these artifacts were uncovered during the recent and ongoing archaeological excavations at the site. In layer *gic*, the frequency of seashells is notably higher compared to *rsa’*, prompting future studies to explore the potential correlation between the site’s use and variations in symbolic artifacts. It is important to note that the exclusive use of seashells at Castelcivita does not correlate with chronological proxies, as it is consistent across southern Italian sites from different Aurignacian phases^76^. For instance, at the site of Cala, which techno-typologically aligns with the EA and is dated to after the CI super-eruption, only seashells were recovered^119^.

Considering these diverse lines of evidence, it appears that EA-like assemblages in Italy are culturally less homogeneous than the preceding PA, revealing intricate processes of regionalization stemming from a shared basal adaptation^31^ common to all Aurignacian foraging groups. In this framework, the predominant inter-regional cultural trend visible in the Aurignacian indicates an emergent tendency^120^ toward the independence of bladelet production^121^. Conversely, the adoption of carinated technology did not coincide with the synchronous adoption of other cultural traits in the realm of personal ornaments and bone tools. This observation emphasizes that the PA–EA technological shift did not entail significant processes of population turnover or a high degree of population interconnectivity^122^. The scenario proposed by Anderson and colleagues^123^ aligns well with this context. The authors view the PA as a pioneering—though not the earliest according to recent findings—phase of *Homo sapiens* dispersal across Europe, followed by a period of geographic stabilization and the emergence of more distinct regional variants. This reconstruction not only elucidates the delayed onset of the PA in southern Italy but also addresses the significant regional variability observed when comparing different geographic regions of Europe^124^.

Future research should delve deeper into investigating the role of varying demographic patterns in triggering regional cultural changes^125^, as well as the extent of cultural transmission processes at different geographic scales, contributing to the roughly contemporaneous technological shifts identified by archaeologists. Testing null models of isolation-by-distance^126^, for instance, could shed light on the processes behind the regionalization of the *gic*–*ars* assemblages when correlated with different demographic simulations and varying degrees of population interconnectivity. It is noteworthy that Castelcivita is positioned at the southern end of the Aurignacian geographic distribution. The distance from other core areas might have influenced the degree of network connectivity among foraging groups settled in the region. Modeling paleo-demography in the Paleolithic poses a considerable challenge, but scholars have been able to demonstrate a strong correlation between population size, network connectivity, and artifact diversity^127^. In this context, we argue that the local development of the miniaturized retouched bladelet type suggests the existence of a local population of sufficient size to initiate innovation and vertical transmission processes that endured long enough to become archaeologically visible.

Another research question deserving exploration concerns the processes underlying the success of carinated technology during the Aurignacian. The gradual replacement of platform cores by carinated cores may be linked to the mobility of foraging groups^121^. Carinated technology allows for the production of standardized bladelets with minimal raw material loss and fewer initialization and maintenance operations compared to platform cores^85,86^, offering a generalized advantage effective in various geological and environmental conditions. Despite raw material selection and transport remaining relatively stable at Castelcivita, the *gic*–*ars* assemblages are characterized by a marked miniaturization^128^ across all components of lithic technology. This strategy might have resulted in a more economical use of raw materials^129^. In Italy, carinated technology is more markedly associated with regions where foragers relied on low-quality raw materials and/or small-sized pebbles^38,119, 130^, suggesting that inter-regional differences in the frequency of carinated cores may also be influenced by the quality and abundance of raw materials. Delving into this research question may also help explain why, in certain regions such as northern Italy, the PA–EA shift is either not evident^27,32^ or occurs later^16,17^ than at Castelcivita. This discussion, however, should be deferred until new dating frameworks and comprehensive assessments of the integrity of lithic assemblages are thoroughly addressed for all sites.

Planned research in the context of southern Italy will help determine whether the regional signal observed in layers *gic*–*ars* at Castelcivita persisted throughout the entire EA duration or if certain elements, such as the miniaturized retouched bladelets, represented short-lived innovations. Two significant candidates for addressing this question are the sites of Cala and Paglicci, both of which feature a stratigraphic sequence that postdates the CI super-eruption. The combined study of these sites will also allow for an assessment of whether and at which point in time cultural transmission processes facilitated the spread of split-based antler points to these southern regions. Confirming this step-like development process would ultimately support our expectation that the shift to the EA did not involve the uptake of the complete behavioral package from its emergence and that underlying demographic dynamics influenced the degree of innovation and transmission mechanisms.

### Concluding remarks

In conclusion, our study emphasizes the need for future research to move beyond monocausal explanations of cultural change, providing new high-resolution data on neglected regions of Europe to better contextualize the development of the Aurignacian. The stratigraphic sequence of Grotta di Castelcivita elucidates the reasons behind the success of the Aurignacian across a broad geographic and temporal spectrum compared to the earlier, more geographically constrained Early Upper Paleolithic technocomplexes. Furthermore, it demonstrates the challenges of investigating cultural transitions at different spatial and temporal scales. The rapid technological shift detected at the site does not neatly align with, and may poorly correlate to, patterns of continuity and discontinuity across other archaeological and environmental factors. Separating the emergent trend that leads to the development of the EA and the adaptation of foraging groups to local settings will require the design of interdisciplinary studies and the expansion of archaeological investigations to nearby sites, fully appreciating the regional signal of the Aurignacian in southern Italy and its long-term development. As rigorously discussed by S. Kuhn, “[…] local and global may have quite different dynamics and require quite different kinds of explanations”^120^. By delving into this research path, a more complex picture of the social organization of human groups in the Upper Paleolithic will emerge.

## Methods

### Attribute analysis and reduction sequence analysis

In this study, we have adopted a comprehensive approach, smoothly integrating various methods to analyze lithic assemblages. Our main focus has been on the production of blades and bladelets across the Aurignacian sequence at Castelcivita. We combined attribute analysis^100,131, 132^ with reduction sequence analysis^133–136^ following the framework set by several other studies^22,27,84, 137–139^. This combined approach allowed us to record and analyze a wide array of discrete and metric attributes on individual lithic artifacts, facilitating the reconstruction of core reduction sequences and enabling comparisons between assemblages. The classification of raw materials was conducted based on macroscopically observable surface features and, when applicable, the type of cortex^132^. Cores were classified based on the specific production objective and the configuration of the striking platform(s) in relation to the flaking surface(s). The primary classification followed Conard and colleagues^140^, while further sub-classifications were applied to platform blade and bladelet cores, following the system proposed by Falcucci and Peresani^82^. The classification and technological descriptions of carinated cores were based on the comprehensive works presented in Le Brun-Ricalens and colleagues ^141^ and the pivotal research of Bon^23^. All laminar cores were measured along their technological axis, following the method outlined by Lombao and colleagues^24^. This implies that the length was measured along the axis of the flaking surface. Consequently, the maximum linear dimensions may differ from the technological dimensions, especially in the case of carinated cores. For bipolar cores, we included all artifacts displaying clear traces of the bipolar technique on anvil, following archaeological and experimental studies^72,73, 98, 142,143^. We excluded from this category those blanks that exhibited only minor evidence of the bipolar technique, such as short and hinged scars covering a small portion of the artifact. Instead, these were classified as scaled pieces and included in the tool category. Cortex coverage, on both cores and tools, was assessed using five categories (0%, 1–33%, 33–66%, 66–99%, and 100%), enabling comparisons between layers.

For tool typologies, we employed a revised and simplified version of some of the most widely used Upper Paleolithic typologies for classifying tools, drawing from the works of de Sonneville-Bordes^87^ and Demars and Laurent^81^. Carinated cores that could also be categorized as endscrapers or burins, depending on the location of the flaking surface, along with burin cores, were additionally classified as tools. Burins were classified as cores when exhibiting multiple bladelet negatives oriented along the longitudinal axis of the blank and featuring a prepared plain striking platform. This approach allowed us to draw comparisons and identify similarities with other previously published Aurignacian assemblages.

Blades and bladelets were categorized based on the metric boundary established by Tixier^144^, defining a bladelet as a laminar blank with a width value below 12 mm. While we acknowledge that this size cut-off is a simplification used for inter-site comparisons, it efficiently captures the essential aspects of Aurignacian lithic production and modification^25^. It is important to note that blades and bladelets are typically defined as blanks with a length at least twice their maximum width. However, it became apparent during the initial scrutiny of materials that this definition could not be rigidly applied at Castelcivita. Several small, regular, and standardized blanks had a length-to-width ratio just slightly below 2. These artifacts closely resembled bladelets produced from carinated cores^85,86^. To avoid adding unnecessary complexity to our results, we chose to classify these artifacts as bladelets, while keeping an additional sub-classification (i.e., flakelets) in the dataset.

The recorded discrete and metric attributes provided us with valuable insights into the knapping technique, the technological organization of stone tool production, and the metric variations within and among assemblages. For recording metric attributes, we utilized a plastic digital caliper which had a precision of 0.2 mm and a resolution of 0.1 mm. A range of discrete attributes was documented on individual lithics to describe the direction and orientation of removals, the shape of platforms and the presence of butts and lips, the profile curvature and twisting of blanks in profile view, and the morphologies of the blanks and distal ends in dorsal view. Retouched tools were excluded from both the metric analysis and the quantification of blank outline and distal end morphologies. Likewise, fragmented blanks were not considered in the morphometric analysis. Statistical assessments of size variability were performed using non-parametric tests such as Wilcoxon and Kruskall-Wallis. To minimize the risk of type 1 errors, we applied the Holm-Bonferroni sequential correction^145^. Statistical analysis was performed on discrete attributes using Pearson’s chi-squared or Fisher’s exact tests. Fisher’s exact test was chosen over Pearson’s test when the total number of available observations was below 1,000, aiming to minimize the risk of violating the assumptions of the test.

### 3D scanning and multivariate analysis of cores

We used an Artec Space Spider (Artec Inc., Luxembourg) to create 3D meshes of all discarded cores at the site, as well as numerous retouched tools, complete blades, and other blanks important for reconstructing the core reduction sequence. In addition to obtaining quantitative data, our objective was to establish an extensive repository of the Aurignacian lithics from Castelcivita. This repository is currently accessible on Zenodo under CC-BY-4.0^80^. Besides the standard metric (e.g., linear dimensions, length, and width of last removals) and discrete (e.g., shape of the striking platform, flaking direction, cortex coverage, blank production) attributes, we quantified two important attributes from the cores’ 3D meshes. Volumes of all cores were calculated using the R package *Rvcg*^146^. The striking angles of blade and bladelet cores were determined in Meshlab^147^ using the Virtual Goniometer plugin^148^. The angles were measured in three distinct areas of the core, namely at locations where the striking platform intersected a successful removal visible on the flaking surface. The mean of these measurements was used for subsequent statistical analysis. To explore core variability and reduce data dimensionality, we employed Principal Component Analysis (PCA) using five quantitative variables: 3D volume, flaking surface length, mean striking angle, flaking surface length to core thickness ratio, and flaking surface length to core width ratio. We prioritized flaking surface length over core length to examine the relationship between the active core area and the geometric orientation of the raw material. Our goal was to identify patterns of technological change between the lowermost layer *rsa’* and *gic*, and to assess the contribution of technological and morphometric features in classifying core types without relying on prior qualitative classifications. To maintain consistency, we excluded layer *ars* from the PCA due to the limited number of cores recovered. Similarly, we omitted cores in the initial stages of reduction as well as core shatters, focusing on identifying patterns of variability among cores discarded after the successful production of laminar blanks. The PCA and interpretation of its outputs were performed using the *FactoMineR*^149,150^ and *factoextra*^151^ R packages. Finally, we used the open-source software *Artifact3-D* to create the views of the 3D models of the cores and tools used in the paper’s figures^152^.

### 2D geometric morphometrics

Quantifying discrete attributes related to the shape of blanks can be challenging as they often rely on descriptors that are subjective and influenced by the analyst’s experience and research background^153^. Various studies in the field of geometric morphometrics (GM) have shown that shape analysis is a crucial component for exploring lithic technological variability and understanding design space constraints in retouched tools ^25,154–160^. To enhance the objectivity of this study, we chose to employ Elliptic Fourier Analysis (EFA)^161^ on both unmodified and retouched bladelets. EFA has been widely used in the analysis of 2D shapes of lithic artifacts, both in dorsal view^155,157,158,162, 163^ and in cross-section ^90,164^. To conduct this analysis, we took high-resolution photos of all complete bladelets using a digital camera. We then extracted the artifacts’ outlines using the open-source software *DiaOutline*^165^, which allows for the automatic extraction of outlines from closed shapes and the export of 2D coordinates in .txt format. Bladelets from *ars* were not included in this study due to the limited number of complete artifacts, particularly tools, retrieved from this layer. We imported the raw coordinate data into R for the 2DGM analysis, using the *Momocs* package^166^. Before performing EFA, we centered, scaled, and rotated the outlines. EFA was conducted using the harmonics that captured 99.9% of the cumulative harmonic power, which equated to 23 harmonics. This analysis allowed us to investigate the mean shape of bladelets across the different layers and to run a PCA on the harmonic coefficients to assess shape differences. All raw outlines, R script, and generated datasets are available for download in the associated research compendium. To explore the mean shape variability between *gic* and *rsa’*, as well as across different groups of tools, we employed a non-parametric MANOVA (i.e., PERMANOVA). We conducted the test using 10,000 repetitions in the *vegan* R package^167^ following Matzig and colleagues^155^. We then utilized the *pairwiseAdonis* package^168^ to calculate pairwise distances using Euclidean distance. For the PERMANOVA, we included only the principal components that accounted for 95% of the explained variance, which amounted to 8 components. Due to sample size, we combined bladelets with inverse and alternate retouch into a single group to allow for a more accurate comparison after confirming the absence of differences between the two retouch types.

### R programming and reproducibility

We performed data manipulation, visualization, and statistical analysis in R v.4.3.1^78^ and RStudio^77^, using several packages for statistical analysis. To enhance the reproducibility of this study, we created a research compendium that comprises all datasets and scripts. This compendium includes detailed explanations of the steps required to execute and reproduce the analyses, and it can be accessed through Zenodo^79^. We utilized the *renv* package^169^ to establish a reproducible environment, enabling the reuse of our code and workflow. The Supplementary Information html file associated with this paper was generated in R Markdown^170^. In addition to the packages mentioned in the previous sections, we utilized the *Tidyverse* packages for data manipulation and visualization^171^, *janitor* for constructing frequency tables^172^, and *Rstatix* for univariate statistics^173^.

### Supplementary Information

Supplementary Information.

## Data Availability

The datasets generated and analyzed in the current study are available in the associated research compendium available on Zenodo: https://doi.org/10.5281/zenodo.10639552. The repository includes the R scripts to reproduce all results and figures of the study, as well as the outline coordinates used in the geometric morphometrics study. Furthermore, all 3D models of cores, core-tools, and tools are published in Zenodo: https://doi.org/10.5281/zenodo.10631389.
