# Supplementary material for: A pre-Campanian Ignimbrite techno-cultural shift in the Aurignacian sequence of Grotta di Castelcivita, southern Italy
Source: Sci Rep. 2024 Jun 4;14:12783. doi: 10.1038/s41598-024-59896-6 (PMC11150382; doi:10.1038/s41598-024-59896-6)
Supplement: Supplementary file 1 — Supplementary Information. [file 41598_2024_59896_MOESM1_ESM.html]

 

 

 

 
 
 


 
 


 Supplementary Information for ‘A pre-Campanian Ignimbrite techno-cultural shift in the Aurignacian sequence of Grotta di Castelcivita, southern Italy’ 

 
 
 
 
 
 
 
 
 
 
 
 
 
 
 
 

 

 
 


 


 

 

 


 


 

 


 


 
 
 
 
 
 

 


 


 Supplementary Information for ‘A
pre-Campanian Ignimbrite techno-cultural shift in the Aurignacian
sequence of Grotta di Castelcivita, southern Italy’ 
 Armando Falcucci, Simona Arrighi, Vincenzo Spagnolo,
Matteo Rossini, Owen Alexander Higgins, Brunella Muttillo, Ivan Martini,
Jacopo Crezzini, Francesco Boschin, Annamaria Ronchitelli, Adriana
Moroni 
  armando.falcucci@uni-tuebingen.de  (Armando Falcucci,
corresponding author) 

 


   
 
 The site 
 
 Grotta di Castelcivita and the Aurignacian stratigraphic
sequence 
 Grotta di Castelcivita (hereafter Castelcivita; Salerno, Campania,
Southern Italy) is a cave site situated at an elevation of 94 meters
above sea level, nestled at the base of the Alburni massif, near the
right bank of the Calore River (40.49563600N, 015.20922177E; see Fig.
1). This cave is part of a karst system that spans two primary levels,
comprising an extensive network of tunnels and chambers, with a total
length exceeding 5 kilometers (Cafaro et al., 2016). Systematic
excavations were first conducted from 1975 to 1988, led by P. Gambassini
of the Research Unit of Prehistory and Anthropology at the University of
Siena (Gambassini, 1997). Subsequently, fieldwork was resumed in 2015 by
the same research unit, under the direction of A. Ronchitelli and A.
Moroni, and continuing to the present year, in collaboration with the
“Soprintendenza Archeologia, Belle Arti e Paesaggio per le province di
Salerno e Avellino”. 
 The archaeological deposit is situated at the actual cave entrance
and presently covers an excavated area of 35 square meters, of which
Gambassini excavated 14 in the last century. Before the systematic
investigations in 1975, nearly 6 square meters were excavated by looters
(see Fig. 3b–d), leaving a deep large pit down to the base of the
anthropogenic sequence. The destination of the archaeological materials
originating from this area is presently unknown. The archaeological
sequence spans a depth of 3.4 meters and contains multiple layers
attributed to the late Mousterian ( cgr ,  gar ,
lower- rsi ), the Uluzzian (upper- rsi ,  pie ,
 rpi , and  rsa’‘ ), and the Aurignacian ( rsa’ ,
 gic , and  ars ; see Fig. 3a). Layer  ars  is
overlaid by a decimeter-thick layer of tephra (Giaccio et al., 2008),
that corresponds to the well-known Campanian Ignimbrite (CI; Fedele et
al., 2004), one of the largest Late Quaternary explosive events and an
example of super-eruption (Sparks et al., 2005). This event was recently
dated to 39.85 ± 0.14 ka BP (Giaccio et al., 2017) and originated from
the largest eruption of the Phlegrean Field caldera. Fallout deposits
connected with this event were recognized in many continental, marine,
and cave successions of Europe and Asia and, consequently, it is one of
the most important temporal and stratigraphic markers of western Eurasia
(d’Errico & Banks, 2015). This tephra layer in the succession of
Castelcivita represents a  terminus ante quem  for the site’s
last human occupations. Layer  CI  is in turn overlaid by a
multi-layered flowstone with embedded thin layers of volcanic ashes that
seal the entire stratigraphic sequence. These thin layers were probably
connected to the surficial reworking of CI deposits, subsequently
redeposited within the cave. 
 In this paper, our focus lies on the Aurignacian sequence, which
follows the last Uluzzian layer  rsa’’ , dated to 41.9–40.6 ky
cal BP (Douka et al., 2014; Wood et al., 2012). The Aurignacian cultural
sequence is thus well-constrained chronologically between the last
Uluzzian and the CI (39.85 ± 0.14 ka BP). This suggests that layers
 rsa’ – ars  accumulated over a relatively short period,
likely just a few centuries (Giaccio et al., 2008). Castelcivita is,
therefore, together with Grotta Paglicci in Gargano (Palma di Cesnola,
2004), a unique case study to investigate the initial stages and early
development of the Aurignacian in southern Italy. 
 The stratigraphic succession of Castelcivita was further revisited in
2020 by I. Martini, adopting a facies analysis-based approach. Layer
 rsa’  is made of fine-grained reddish sediments dominated by
silt and sand with scattered small limestone debris (2-4 cm in size).
The limestone debris results from the cave’s roof degradation, while the
sandy sediments are linked to infiltration processes (sensu Martini et
al., 2018; Martini et al., 2021) from the outer area. This layer is
12–15 cm thick and, generally, in direct continuity with the Uluzzian
from layer  rsa’’ , except for a few discontinuous thin sterile
sandy lenses, separating the two levels in some areas. The subsequent
layer,  gic , has a thickness of around 20 cm and is well
distinguishable from  rsa’  due to its yellowish color and the
presence of extensive concretions. Finally, layer  ars  was
excavated over a very limited area because the upper sequence was almost
completely disrupted during the construction of an artificial entrance
for visitor access to the cave. The sediment composition is similar to
that of  rsa’ , primarily sandy but with a lighter color (i.e.,
orange). 
 The excavations revealed a few features across the Aurignacian
sequence. In the area excavated by Gambassini, a part of an extended
surface with fire was identified at the base of layer  gic , in
square H14 between 80 and 90 cm deep. The remaining portion of this
surface was brought to light in 2016 in square H15, thus attesting to
the presence of a large or two smaller adjacent fireplaces
(Supplementary Fig. S1). The fireplace/s, around 2x1 m in total,
was/were composed of a layer of ash (above) and a layer of charcoal
(below) and was/were lying directly on the surface of the ground,
partially in contact with the underlying layer rsa’. Gambassini’s
excavations did not report any traces of fireplaces in layer
 rsa’ , except for a cluster of charcoal found in square H13.
However, recent investigations have also revealed similar features to
 gic  in this layer. 
 The excavations conducted between 1975 and 1988 employed a
stratigraphic method. Findings were meticulously documented using a grid
coordinate system, which consisted of square meters further subdivided
into 50x50 cm sectors. Layers were systematically excavated using 10 cm
deep spits, further divided into 5 cm sub-spits. Excavators paid close
attention to identified discontinuities within each spit, allowing them
to follow sloping deposits and anthropogenic features accurately. All
archaeological findings were precisely assigned to a specific square,
sector, layer, and sub-spit. Furthermore, a significant portion of the
materials were spatially documented in three dimensions using elevation
(Z) coordinates. All sediments were carefully dried sieved and
subsequently subjected to wet sieving with a 1 mm mesh. Further
screening was then performed to isolate and categorize all finds. 
  
 Figure S1.  Plan view of the excavation of layer
 gic  (spit 8) depicting evidence of the large anthropogenic
feature/s (in black) identified in squares H14 and H15. The other
excavated squares are colored yellow. Please, refer to the provided
legend for interpreting the drawing. 
   
 
 
 The environmental and ecological setting 
 The environmental and ecological setting of Castelcivita was
comprehensively examined by studying large and small mammals, avifauna,
ichtyofauna, anthracological remains, and the sedimentary composition of
the sequence (see papers in Gambassini, 1997). The evidence gathered
highlights significant changes during the transition from  rsa’ 
to  gic . Layer  rsa’  contains a fauna assemblage that is
notably similar to the last Uluzzian of layer  rsa’’ , marked by
the predominance of horses and a significant presence of  Microtus
arvalis/agrestis  and  Microtus (Terricola) savii . Even if
rare, taxa related to milder or more humid conditions are still present
(e.g. fallow deer,  Apodemus  sp. or  Eliomys  sp.). The
avifauna, on the other hand, is characterized by a high frequency of
steppe grassland species, particularly those associated with rocky
environments. These findings, alongside the sedimentary composition of
the layer, collectively suggest relatively cold and arid conditions,
featuring open environments with sparse woodlands. Climatic features do
not look to be extreme, as indicated by the presence of taxa more
related to Mediterranean conditions. In layer  gic , there is a
discernible shift in climate towards a more humid, cold-temperate
environment. This is accompanied by a reduction in the presence of the
horse and an increase in red deer, roe deer, and chamois. Additionally,
there is a slight rise in forest and water bird species (Fiore et al.,
2020; Gambassini, 1997). Finally, layer  ars  records a new cold
phase, evident particularly in the composition of anthracological
remains dominated by  Pinus  and  Betula . 
 Integrated studies are ongoing to establish a correlation between the
local environmental signal detected across the stratigraphic sequence
and supra-regional climatic changes. Some evidence seems to indicate
that Heinrich Event 4 (H4) commenced slightly before the deposition of
the CI tephra (Badino et al., 2020; Lowe et al., 2012; Margari et al.,
2009; Wulf et al., 2018; Wutke et al., 2015). In northern Italy, such as
at Fumane Cave, H4 has been identified based on micro- and macro-fauna
evidence (López-García et al., 2015). Marín-Arroyo et al. (2023), for
instance, associated the reduced frequency of red deer in layer
 D3  with the onset of H4. Although the fauna assemblage
composition may reflect hunting choices made by Aurignacian foragers
(Discamps et al., 2011), significant oscillations in the frequency of
red deer and horse at Castelcivita likely indicate changes in the
surrounding environment of the cave. It is noteworthy, however, that red
deer exhibit great ecological plasticity and can adapt to steppe
environments, though they prefer patchy wooded areas (Discamps et al.,
2011). At Castelcivita, two cold phases could be associated with the
onset of H4. The first is linked with the driest phase of the sequence
detected in the late Uluzzian ( rsa’’ ) and the Protoaurignacian
( rsa’ ) layers. Despite internal climatic oscillations being
recorded across the period of H4 (Margari et al., 2009; Skinner &
Elderfield, 2007), the amelioration detected in layer  gic  is
rather marked and suggests that  rsa’’ – rsa’  accumulated
more likely during the short-lived cold stadial GS 9/10, with layer gic
corresponding to the GI 9 (Andersen et al., 2006; Rasmussen et al.,
2014; Svensson et al., 2008). In this framework, H4 would only start in
the uppermost layer  ars . 
 While an accurate taphonomic study is still in progress, we can state
that humans played a significant role in the accumulation of mammal
bones in the Aurignacian layers. Notably, evidence of carnivore
activity, such as gnawing marks, is relatively low in comparison to the
preceding Mousterian and Uluzzian (Romandini et al., 2020). This pattern
slightly differs for birds, where there are fewer anthropogenic
modifications, but several marks produced by carnivores. It is important
to consider that the reduced exploitation of birds and fishes compared
to the Uluzzian layers may be linked to differences in site use, a
factor that warrants further exploration (Fiore et al., 2020). 
   
 
 
 
 Results 
 
 Raw material variability 
 The most prevalent raw material at Castelcivita is the local
fine-grained chert, with comparable frequencies (approximately 90%)
observed across the sequence. In our studied sample, quartzite,
radiolarite, and coarse-grained chert were utilized at relatively low
frequencies (Tables S1 and S2). Raw materials could be procured in the
form of large-sized blocks from primary or sub-primary sources in the
vicinity of the cave, as well as river pebbles, which were locally
available along the riverbed of the Calore stream (Gambassini, 1997;
Rossini et al., 2022). Unworked raw material blocks were not uncovered
within the excavated area. Both tested (n = 2) and initial (n = 17)
cores suggest that foraging groups opted to transport thick flakes and
chunks, likely having shattered the raw material blocks away from the
site. 
   
 
 
 
 
 
 
Layer
 
 
 
 
 
 
 
 
 
 
Raw material
 
 
ars
 
 
gic
 
 
rsa’
 
 
 
 
Coarse-grained chert
 
 
3 (3.0%)
 
 
17 (1.4%)
 
 
34 (2.6%)
 
 
 
 
Fine-grained chert
 
 
89 (88.1%)
 
 
1,125 (92.5%)
 
 
1,193 (91.5%)
 
 
 
 
Limestone
 
 
1 (1.0%)
 
 
0 (0.0%)
 
 
0 (0.0%)
 
 
 
 
Quartzite
 
 
6 (5.9%)
 
 
32 (2.6%)
 
 
59 (4.5%)
 
 
 
 
Radiolarite
 
 
2 (2.0%)
 
 
42 (3.5%)
 
 
18 (1.4%)
 
 
 
 
Total
 
 
101 (100.0%)
 
 
1,216 (100.0%)
 
 
1,304 (100.0%)
 
 
 
 
  Table S1.  Distribution of blanks and tools
categories sorted according to raw material type. Rounded percentages
are given in brackets. 
   
 
 
 
 
 
 
Layer
 
 
 
 
 
 
 
 
 
 
Raw material
 
 
ars
 
 
gic
 
 
rsa’
 
 
 
 
Coarse-grained chert
 
 
0 (0.0%)
 
 
1 (1.1%)
 
 
10 (8.1%)
 
 
 
 
Fine-grained chert
 
 
8 (88.9%)
 
 
89 (94.7%)
 
 
105 (85.4%)
 
 
 
 
Limestone
 
 
1 (11.1%)
 
 
1 (1.1%)
 
 
2 (1.6%)
 
 
 
 
Quartzite
 
 
0 (0.0%)
 
 
1 (1.1%)
 
 
5 (4.1%)
 
 
 
 
Radiolarite
 
 
0 (0.0%)
 
 
2 (2.1%)
 
 
1 (0.8%)
 
 
 
 
Total
 
 
9 (100.0%)
 
 
94 (100.0%)
 
 
123 (100.0%)
 
 
 
 
  Table S2.  Distribution of core and core-tool
categories sorted according to raw material type. Rounded percentages
are given in brackets. 
   
 
 
 Flake production and bipolar technology 
 Flakes were produced in all analyzed assemblages using platform,
multidirectional, and bipolar strategies (Table S3). The latter are
particularly attested in  rsa’  and sharply decrease in the
following layer  gic  (Fig. S2). 
   
 
 
 
 
Layer
 
 
Bipolar
 
 
Platform
 
 
Multidirectional
 
 
Shatter
 
 
Total
 
 
 
 
 
 
ars
 
 
1 (50.0%)
 
 
1 (50.0%)
 
 
0 (0.0%)
 
 
0 (0.0%)
 
 
2 (100.0%)
 
 
 
 
gic
 
 
15 (41.7%)
 
 
2 (5.6%)
 
 
12 (33.3%)
 
 
7 (19.4%)
 
 
36 (100.0%)
 
 
 
 
rsa’
 
 
48 (71.6%)
 
 
9 (13.4%)
 
 
7 (10.4%)
 
 
3 (4.5%)
 
 
67 (100.0%)
 
 
 
 
Total
 
 
64 (61.0%)
 
 
12 (11.4%)
 
 
19 (18.1%)
 
 
10 (9.5%)
 
 
105 (100.0%)
 
 
 
 
  Table S3.  Core types associated with the production
of flakes across the studied assemblages. The category  Shatter 
contains fragments that do not retain any evidence for laminar
productions. The table does not include tested cores (n = 2) as the
production objective could be not assessed. Rounded percentages are
given in brackets. 
   
  
 Figure S2.  Bipolar cores found in layers  gic 
(a) and  rsa’  (b-f). The numbers shown after the letters are
from the dataset created by one of us (AF). In both the dataset and the
3D model repository, they are prefixed with “CTC”, which is the common
abbreviation for the site. 
   
 Most of the bipolar cores have two opposed striking platforms (Table
S4) and in a few cases cores were rotated to carry on the blank
production from orthogonal platforms. One of the most recurrent features
of bipolar cores is the presence of at least a dihedral platform, which
likely results from the disintegration of the core’s platform after
several bipolar strikes (Arrighi et al., 2020; Peresani et al., 2019).
Given that this platform type is usually found in only one side of the
core, it has been supposed that this area was in contact with the anvil
throughout the reduction (Arrighi et al., 2020). Scars of negatives can
be typed in most cases as flakes and many show stepped or hinged
terminations. Only in a limited number of cases bladelet-like scars are
visible alongside with flake removals (n = 9, 7 in  rsa’  and 2
in  gic ). Overall, there is no clear intention to produce
bladelets in this class of cores and evidence of bipolar knapping is
extremely low among the analyzed laminar blanks. Likewise, tools made on
bipolar blanks are extremely rare ( rsa’  = 6,  gic  = 1,
and  ars  = 1) and never associated with tool types such as
endscrapers, burins, and retouched blades or bladelets . 
   
 
 
 
 
 
 
Number of platforms
 
 
 
 
 
 
 
 
 
 
 
 
 
 
Layer
 
 
1
 
 
2
 
 
3
 
 
4
 
 
Total
 
 
 
 
ars
 
 
0 (0.0%)
 
 
1 (100.0%)
 
 
0 (0.0%)
 
 
0 (0.0%)
 
 
1 (100.0%)
 
 
 
 
gic
 
 
1 (6.7%)
 
 
13 (86.7%)
 
 
0 (0.0%)
 
 
1 (6.7%)
 
 
15 (100.0%)
 
 
 
 
rsa’
 
 
5 (10.4%)
 
 
39 (81.2%)
 
 
4 (8.3%)
 
 
0 (0.0%)
 
 
48 (100.0%)
 
 
 
 
Total
 
 
6 (9.4%)
 
 
53 (82.8%)
 
 
4 (6.2%)
 
 
1 (1.6%)
 
 
64 (100.0%)
 
 
 
 
  Table S4.  Number of striking platforms recorded on
bipolar cores in the studied assemblages. Rounded percentages are given
in brackets. 
   
 A comparison of the 3D volume of freehand and bipolar cores in both
 rsa’  and  gic  shows that bipolar cores have
significantly lower values (Fig. S3), suggesting that this reduction
technique allowed knappers to maximize blank production and exhaust most
of the volume available. At the same time, the use of this technique can
result in the split of the core in two or more bipolar shatters that are
likely to be classified as bipolar cores due to the difficulty in
finding a clear separation between them. This would also explain the
high frequency of bipolar cores compared to freehand flake cores.
Likewise, bipolar technique could also be used in an advanced stage of
reduction to maximize blank production. In this regard, bipolar cores
preserve less often cortical remains compared to platform and
multidirectional flake cores (Table S5). 
   
  
 Figure S3.  Comparison of the volume values of freehand
(i.e., platform and multidirectional) and bipolar cores in  gic 
and  rsa’ . Layer  ars  is not displayed as only one
bipolar core is available. The figure displays also the results of the
Wilcoxon tests comparing the volume values of freehand and bipolar cores
within each layer. 
   
 
 
 
 
 
 
Core type
 
 
 
 
 
 
 
 
 
 
 
 
Cortex
 
 
Bipolar
 
 
Platform
 
 
Multidirectional
 
 
Shatter
 
 
 
 
0%
 
 
48 (75.0%)
 
 
2 (16.7%)
 
 
6 (31.6%)
 
 
4 (40.0%)
 
 
 
 
1-33%
 
 
10 (15.6%)
 
 
6 (50.0%)
 
 
7 (36.8%)
 
 
2 (20.0%)
 
 
 
 
33-66%
 
 
2 (3.1%)
 
 
4 (33.3%)
 
 
5 (26.3%)
 
 
4 (40.0%)
 
 
 
 
66-99%
 
 
4 (6.2%)
 
 
0 (0.0%)
 
 
1 (5.3%)
 
 
0 (0.0%)
 
 
 
 
Total
 
 
64 (100.0%)
 
 
12 (100.0%)
 
 
19 (100.0%)
 
 
10 (100.0%)
 
 
 
 
  Table S5.  Percentage of cortex coverage recorded on
flake cores considering all layers as a single group. Rounded
percentages are given in brackets. 
   
 
 
 Initialization and configuration of blade and bladelet cores 
 Despite the advanced stage of reduction resulting in the discard of
most cores, we managed to identify the blank types selected for knapping
laminar blanks. Knappers typically selected block chunks, pebbles, and
thick flakes for laminar production (Table S6). Striking platforms are
consistently plain and were created by either using core tablets or
positioning them on a ventral face when a flake was chosen. Faceted
platforms are on the other hand absent. The main operations observed on
initial cores primarily involved the decortication and shaping of the
longitudinal and transversal convexities, typically executed through
unidirectional strategies. Blank production often began by removing
fully cortical or dihedral blanks, making use of sharp natural angles.
Primary crests are also documented, and in most cases, they are
one-sided, indicating that only one flank of the core was shaped with
orthogonal removals. Crested blanks are relatively rare in  gic 
(n = 3) and  ars  (n = 1), while they are more common in
 rsa’  (n = 25). 
   
 
 
 
 
 
 
Selected blank
 
 
 
 
 
 
 
 
 
 
 
 
 
 
 
 
 
 
 
 
Layer
 
 
Angular debris
 
 
Blade
 
 
Block
 
 
Core fragment
 
 
Flake
 
 
Pebble
 
 
Undetermined
 
 
Total
 
 
 
 
ars
 
 
0 (0.0%)
 
 
0 (0.0%)
 
 
1 (14.3%)
 
 
0 (0.0%)
 
 
4 (57.1%)
 
 
1 (14.3%)
 
 
1 (14.3%)
 
 
7 (100.0%)
 
 
 
 
gic
 
 
3 (5.2%)
 
 
1 (1.7%)
 
 
4 (6.9%)
 
 
1 (1.7%)
 
 
36 (62.1%)
 
 
4 (6.9%)
 
 
9 (15.5%)
 
 
58 (100.0%)
 
 
 
 
rsa’
 
 
0 (0.0%)
 
 
2 (3.7%)
 
 
17 (31.5%)
 
 
3 (5.6%)
 
 
14 (25.9%)
 
 
6 (11.1%)
 
 
12 (22.2%)
 
 
54 (100.0%)
 
 
 
 
Total
 
 
3 (2.5%)
 
 
3 (2.5%)
 
 
22 (18.5%)
 
 
4 (3.4%)
 
 
54 (45.4%)
 
 
11 (9.2%)
 
 
22 (18.5%)
 
 
119 (100.0%)
 
 
 
 
  Table S6.  Classification of cores according to the
identified blank used for blade and bladelet productions. The category
 Undetermined  includes all cores that do not retain enough
information to identify the blank selected. Rounded percentages are
given in brackets. 
   
 
 
 Production and maintenance operations on blade and bladelet
cores 
 
 Carinated cores 
 Maintenance operations on carinated cores typically resulted in wide
and convex flakes, often exhibiting bladelet negatives on the dorsal
side. These operations aimed to isolate the flaking surface and maintain
its transversal convexities, while also removing areas of the flaking
surface with hinged removals. Such blanks have been identified in
assemblages characterized by the presence of carinated technology
(Kolobova et al., 2014; Le Brun-Ricalens, 2005). In total, we identified
110 blanks used for maintaining carinated cores. Notably, the majority
of these blanks are from layer  gic  (n = 78, 71%). We compared
the lengths of these blanks with the flaking surface of carinated cores
in both  gic  and  rsa’ , observing a general intra-layer
agreement (Fig. S5). This finding supports the specificity and
stratigraphic attribution of carinated technology to all studied
assemblages. 
   
  
 Figure S4.  Comparison of the length of flaking surfaces
of carinated cores across the studied sequence. The figure displays the
results of the Kruskal-Wallis test and the pairwise comparisons. 
   
  
 Figure S5.  Boxplots showing the distribution of length
values of the flaking surfaces of carinated cores and the length of
blanks identified as belonging to the maintenance of carinated cores.
The figure also displays the results of the Wilcoxon tests comparing
these values within  gic  and  rsa’ . 
   
 
 
 Platform cores 
 Bladelets are the predominant production goal across the sequence.
Notably, independent blade production is only observed in  gic ,
while simultaneous blade-bladelet production is most evident in
 rsa’ . Simultaneous productions can also be inferred from the
presence of blades with visible bladelet scars on their dorsal sides
(Bon & Bodu, 2002). These scars indicate either the detachment of
bladelets during blade reduction sequences (i.e., when the core allowed
for blade production) or the removal of large blanks from bladelet
cores, primarily for maintaining their convexities (Falcucci et al.,
2017). At Castelcivita, 38 out of 42 blades with bladelet negatives
identified relate to maintenance operations on bladelet cores, with the
majority from layer  rsa’  ( 71% , n = 30). This
combined evidence is not surprising when considering that layer
 gic  is primarily defined by the use of carinated technology,
which rarely results in the detachment of maintenance blades (Le
Brun-Ricalens, 2005). 
   
 
 
 PCA of blade and bladelet cores 
  
 Figure S6.  Visualization of the results of the first
and third components of the PCA conducted on laminar cores.
 A  shows a biplot with the contribution of the different
quantitative variables to the first and second components.
 B  and  C  display the distribution of
the studied cores in the PC1 to PC3 space, sorted according to layer
( B ) and core classification ( C ). In
 A ,  FSL  stands for flaking surface length,
 FSL/T  is the ratio between flaking surface length and
thickness,  FSL/W  is the ratio between flaking surface length
and width. The category Narrow/Burin includes narrow-sided cores and
burin cores. Initial cores were excluded from the analysis. 
   
 
 
 
 Morphometric analysis of blades and bladelets 
 In this section, we will explore the morphometric analysis of
complete blades and bladelets to further delve into technological
variability across the sequence. 
 
 Blades 
 The number of available blades is relatively low compared to
bladelets, primarily due to blade production not being a primary goal at
the site. Constraints imposed by locally available raw materials may
have played a role in that. Nonetheless, we have a statistically
suitable sample for conducting a morphometric analysis of layers
 gic  and  rsa’ . In both Layers, blades were produced
using direct marginal percussion (Tables S7-S10 and Fig. S7). The
platforms are generally plain, and their comparable dimensions suggest a
uniform knapping technique. The presence of lipped internal platform
edges and moderately marked bulbs suggests the use of soft hammers,
whether mineral or organic. Blade production is characterized by
predominantly unidirectional sub-parallel removals, with bidirectional
scars being rare. Differences were not found in profile curvature,
profile twisting, and blank shape (Tables S11-S14). On the other hand,
the study of cross-sections suggests that the increased frequency of
trapezoidal and, to a lesser extent, polyhedral shapes are in part to be
linked to the frequent use of blades in  rsa’  for maintaining
bladelet cores (Table S15). Finally, elongation (length to width ratio)
and robustness (width to thickness ratio) ratios remain stable across
 gic  and  rsa’  (Table S16, Figs. S8 and S9), whereas
linear measurements show that blades from  gic  are shorter and
narrower (Table S17 and Fig. S10). 
   
 
 Tables and figures reporting attributes linked to the knapping
technique 
   
 
 
 
 
 
 
Layer
 
 
 
 
 
 
 
 
Platform type
 
 
gic
 
 
rsa’
 
 
 
 
Plain
 
 
33 (76.7%)
 
 
44 (67.7%)
 
 
 
 
Linear/Punctiform
 
 
4 (9.3%)
 
 
7 (10.8%)
 
 
 
 
Other
 
 
5 (11.6%)
 
 
10 (15.4%)
 
 
 
 
Undetermined
 
 
1 (2.3%)
 
 
4 (6.2%)
 
 
 
 
Total
 
 
43 (100.0%)
 
 
65 (100.0%)
 
 
 
 
  Table S7.  Platform types recorded on blades from
 rsa’  and  gic . The category  Other  includes
categories found in low frequencies (e.g., cortical, dihedral, double,
abraded). Linear and punctiform types are grouped in a single category.
A Fisher’s Exact Test reveals no differences between layers ( p 
= 0.78). 
   
 
 
 
 
Layer
 
 
variable
 
 
n
 
 
mean
 
 
sd
 
 
min
 
 
median
 
 
max
 
 
 
 
 
 
gic
 
 
Platform_width
 
 
41
 
 
4.188
 
 
2.683
 
 
0.2
 
 
3.80
 
 
12.5
 
 
 
 
gic
 
 
Platform_thickness
 
 
41
 
 
2.037
 
 
1.576
 
 
0.2
 
 
1.70
 
 
6.4
 
 
 
 
rsa’
 
 
Platform_width
 
 
60
 
 
4.760
 
 
3.517
 
 
0.2
 
 
3.35
 
 
15.8
 
 
 
 
rsa’
 
 
Platform_thickness
 
 
60
 
 
2.188
 
 
1.942
 
 
0.1
 
 
1.50
 
 
8.1
 
 
 
 
  Table S8.  Summary statistics (in mm) of the width
and thickness measurements recorded on blades.  SD  stands for
standard deviation. 
   
  
 Figure S7.  Boxplots showing the distribution of
platform width ( A ) and thickness ( B )
values in  gic  and  rsa’ . The figure is complemented by
the results of the performed Wilcoxon tests, confirming the marked
similarity of these attributes between layers. 
   
 
 
 
 
 
 
Lip type
 
 
 
 
 
 
 
 
 
 
 
 
Layer
 
 
Absent
 
 
Moderate
 
 
Pronounced
 
 
Total
 
 
 
 
gic
 
 
19 (44.2%)
 
 
9 (20.9%)
 
 
15 (34.9%)
 
 
43 (100.0%)
 
 
 
 
rsa’
 
 
32 (49.2%)
 
 
13 (20.0%)
 
 
20 (30.8%)
 
 
65 (100.0%)
 
 
 
 
  Table S9.  Presence and type of lips recorded on
blades. A Fisher’s Exact Test reveals no differences between layers
( p  = 0.87). 
   
 
 
 
 
 
 
Bulb type
 
 
 
 
 
 
 
 
 
 
 
 
Layer
 
 
Absent
 
 
Moderate
 
 
Pronounced
 
 
Total
 
 
 
 
gic
 
 
15 (34.9%)
 
 
23 (53.5%)
 
 
5 (11.6%)
 
 
43 (100.0%)
 
 
 
 
rsa’
 
 
22 (33.8%)
 
 
36 (55.4%)
 
 
7 (10.8%)
 
 
65 (100.0%)
 
 
 
 
  Table S10.  Presence and type of bulbs recorded on
blades. Fisher’s Exact Test reveals no differences between layers
( p  = 1). 
   
 
 
 Tables and figures reporting technological and morphological
attributes 
   
 
 
 
 
 
 
Scar pattern
 
 
 
 
 
 
 
 
 
 
 
 
 
 
Layer
 
 
Unidirectional parallel
 
 
Unidirectional convergent
 
 
Bidirectional
 
 
Other
 
 
Total
 
 
 
 
gic
 
 
24 (55.8%)
 
 
13 (30.2%)
 
 
3 (7.0%)
 
 
3 (7.0%)
 
 
43 (100.0%)
 
 
 
 
rsa’
 
 
28 (43.1%)
 
 
19 (29.2%)
 
 
6 (9.2%)
 
 
12 (18.5%)
 
 
65 (100.0%)
 
 
 
 
  Table S11.  Scar patterns recorded on the blade
assemblages. The  Other  category includes scar patterns found in
low frequencies (e.g., crossed, unidirectional transverse, and
undetermined patterns). The result of a Fisher’s Exact Test reveals no
differences between layers ( p  = 0.34). 
   
 
 
 
 
 
 
Curvature
 
 
 
 
 
 
 
 
 
 
 
 
Layer
 
 
Curved
 
 
Curved slightly
 
 
Straight
 
 
Total
 
 
 
 
gic
 
 
22 (51.2%)
 
 
6 (14.0%)
 
 
15 (34.9%)
 
 
43 (100.0%)
 
 
 
 
rsa’
 
 
32 (49.2%)
 
 
18 (27.7%)
 
 
15 (23.1%)
 
 
65 (100.0%)
 
 
 
 
  Table S12.  Presence and intensity of profile
curvature recorded on complete blades. The result of a Fisher’s Exact
Test reveals no differences between layers ( p  = 0.16). 
   
 
 
 
 
 
 
Torsion simplified
 
 
 
 
 
 
 
 
 
 
Layer
 
 
no
 
 
yes
 
 
Total
 
 
 
 
gic
 
 
29 (67.4%)
 
 
14 (32.6%)
 
 
43 (100.0%)
 
 
 
 
rsa’
 
 
42 (64.6%)
 
 
23 (35.4%)
 
 
65 (100.0%)
 
 
 
 
  Table S13.  Presence of profile twisting recorded on
complete blades. The result of a Fisher’s Exact Test reveals no
differences between layers ( p  = 0.84). 
   
 
 
 
 
 
 
Blank shape
 
 
 
 
 
 
 
 
 
 
 
 
 
 
Layer
 
 
Sub-parallel
 
 
Convergent
 
 
Irregular
 
 
Other
 
 
Total
 
 
 
 
gic
 
 
19 (55.9%)
 
 
2 (5.9%)
 
 
9 (26.5%)
 
 
4 (11.8%)
 
 
34 (100.0%)
 
 
 
 
rsa’
 
 
22 (37.3%)
 
 
9 (15.3%)
 
 
22 (37.3%)
 
 
6 (10.2%)
 
 
59 (100.0%)
 
 
 
 
  Table S14.  External shape recorded on blades. The
category  Other  includes categories found in low frequencies
(e.g., convex, comma-like). A Fisher’s Exact Test reveals no differences
between layers ( p  = 0.27). 
   
 
 
 
 
 
 
Cross-section
 
 
 
 
 
 
 
 
 
 
 
 
 
 
Layer
 
 
Lateral steep
 
 
Polyhedral
 
 
Trapezoidal
 
 
Triangular
 
 
Total
 
 
 
 
gic
 
 
11 (25.6%)
 
 
9 (20.9%)
 
 
11 (25.6%)
 
 
12 (27.9%)
 
 
43 (100.0%)
 
 
 
 
rsa’
 
 
12 (18.5%)
 
 
20 (30.8%)
 
 
30 (46.2%)
 
 
3 (4.6%)
 
 
65 (100.0%)
 
 
 
 
  Table S15.  Cross-section shape recorded on blades. A
Fisher’s Exact Test reveals significant differences between layers
( p  = 0.002). 
   
 
 
 
 
Layer
 
 
variable
 
 
n
 
 
mean
 
 
sd
 
 
min
 
 
median
 
 
max
 
 
 
 
 
 
gic
 
 
Elongation
 
 
34
 
 
2.5
 
 
0.4
 
 
2.0
 
 
2.4
 
 
3.8
 
 
 
 
gic
 
 
Robustness
 
 
34
 
 
3.2
 
 
1.0
 
 
1.6
 
 
3.1
 
 
5.9
 
 
 
 
rsa’
 
 
Elongation
 
 
59
 
 
2.6
 
 
0.5
 
 
2.0
 
 
2.5
 
 
4.0
 
 
 
 
rsa’
 
 
Robustness
 
 
59
 
 
3.3
 
 
1.3
 
 
1.5
 
 
3.0
 
 
7.2
 
 
 
 
  Table S16.  Summary statistics of the elongation
(length to width ratio) and robustness (width to thickness ratio) of
blades.  SD  stands for standard deviation. 
   
  
 Figure S8.  Boxplots showing the distribution of
elongation ( A ) and robustness ( B )
ratios in  gic  and  rsa’ . The figure is complemented by
the results of the performed Wilcoxon tests, confirming the similarity
of these attributes between layers, especially in relation to
robustness. 
   
 
 
 Tables and figures reporting metric attributes 
   
 
 
 
 
Layer
 
 
variable
 
 
n
 
 
mean
 
 
sd
 
 
min
 
 
median
 
 
max
 
 
 
 
 
 
gic
 
 
Length
 
 
34
 
 
36.0
 
 
7.9
 
 
25.0
 
 
36.0
 
 
59.6
 
 
 
 
gic
 
 
Width
 
 
34
 
 
14.6
 
 
2.4
 
 
12.1
 
 
13.6
 
 
20.8
 
 
 
 
gic
 
 
Thickness
 
 
34
 
 
5.0
 
 
1.9
 
 
2.8
 
 
4.6
 
 
10.5
 
 
 
 
rsa’
 
 
Length
 
 
59
 
 
42.5
 
 
10.3
 
 
24.7
 
 
42.7
 
 
65.5
 
 
 
 
rsa’
 
 
Width
 
 
59
 
 
16.4
 
 
4.0
 
 
12.3
 
 
15.7
 
 
31.6
 
 
 
 
rsa’
 
 
Thickness
 
 
59
 
 
5.8
 
 
2.9
 
 
2.0
 
 
4.9
 
 
16.2
 
 
 
 
  Table S17.  Summary statistics of linear dimensions
(length, width, and thickness in mm) recorded on complete blades,
excluding those modified by lateral retouch.  SD  stands for
standard deviation. 
   
  
 Figure S9.  Boxplots showing the distribution of length
( A ), width ( B ), and thickness
( C ) in  gic  and  rsa’ . The figure is
complemented by the results of the performed Wilcoxon tests, showing
that blades from  gic  are shorter and narrower. 
   
 
 
 
 Bladelets 
 In contrast to blades, bladelets exhibit more noticeable variations
across the sequence, and the larger number of artifacts found in
 ars  allows us to include this layer in the comparison. Bladelet
blanks were still detached using direct freehand knapping, but in
 gic  and  ars , the motion appears to have been more
marginal compared to  rsa’ , as visible from the increased
presence of linear and punctiform platforms (Tables S18-S21). Notably,
significant differences in platform width and thickness are observed
between  rsa’  and the upper layers (Fig. S10). Bulbs are more
frequently absent in  gic  and especially  ars , whereas
lipped internal platforms are more frequent. Further experimental work
is required to determine if these differences are related to distinct
knapping techniques used in carinated core reduction. In terms of the
flaking direction recorded on the visible scars of bladelets, it is
almost always unidirectional (Table S22). In gic, reduction pattern is
more frequently convergent, although no differences were identified in
the external morphology and distal ends in dorsal view (Tables S23 and
S24, but see 2DGM analysis). Profiles are straighter in  gic ,
while profile twisting is more common in  rsa’  (Tables S25-S26).
In layers  ars  and  gic , bladelet cross-sections are
often triangular (Table S27), suggesting a preference for a single core
ridge to guide removal (see below). The elongation ratio confirms
significant morphological variability between the upper layers and
 rsa’ , whereas the robustness remains relatively consistent
throughout the sequence (Table S28 Fig. S11). A more detailed
exploration of these morphological aspects through a shape analysis,
including retouched and unretouched specimens, is presented after the
tool analysis. As depicted in Fig. S12, the bladelets recovered in
 gic  and  ars  are smaller in terms of length, width, and
thickness compared to those from  rsa’  (Table S29). The
differences in length values are particularly pronounced and can be
associated with the increased use of carinated technology. 
   
 
 Tables and figures reporting attributes linked to the knapping
technique 
   
 
 
 
 
 
 
Layer
 
 
 
 
 
 
 
 
 
 
Platform type
 
 
ars
 
 
gic
 
 
rsa’
 
 
 
 
Plain
 
 
15 (34.9%)
 
 
199 (35.3%)
 
 
140 (50.5%)
 
 
 
 
Linear
 
 
18 (41.9%)
 
 
252 (44.7%)
 
 
87 (31.4%)
 
 
 
 
Punctiform
 
 
9 (20.9%)
 
 
107 (19.0%)
 
 
32 (11.6%)
 
 
 
 
Other
 
 
1 (2.3%)
 
 
3 (0.5%)
 
 
15 (5.4%)
 
 
 
 
Undetermined
 
 
0 (0.0%)
 
 
3 (0.5%)
 
 
3 (1.1%)
 
 
 
 
Total
 
 
43 (100.0%)
 
 
564 (100.0%)
 
 
277 (100.0%)
 
 
 
 
  Table S18.  Platform types recorded on bladelets. The
category  Other  includes categories found in low frequencies
(e.g., cortical, dihedral, double, abraded). A Pearson’s chi-squared
test reveals significant differences between layers (Chi-squared =
Chi=47.56,  p  < 0.01). 
   
 
 
 
 
Layer
 
 
variable
 
 
n
 
 
mean
 
 
sd
 
 
min
 
 
median
 
 
max
 
 
 
 
 
 
ars
 
 
Platform_width
 
 
43
 
 
1.581
 
 
1.186
 
 
0.1
 
 
1.5
 
 
5.7
 
 
 
 
ars
 
 
Platform_thickness
 
 
43
 
 
0.567
 
 
0.616
 
 
0.1
 
 
0.2
 
 
2.7
 
 
 
 
gic
 
 
Platform_width
 
 
561
 
 
1.580
 
 
1.027
 
 
0.1
 
 
1.6
 
 
7.5
 
 
 
 
gic
 
 
Platform_thickness
 
 
561
 
 
0.484
 
 
0.528
 
 
0.1
 
 
0.2
 
 
4.9
 
 
 
 
rsa’
 
 
Platform_width
 
 
271
 
 
2.060
 
 
1.358
 
 
0.1
 
 
2.0
 
 
10.0
 
 
 
 
rsa’
 
 
Platform_thickness
 
 
271
 
 
0.788
 
 
0.794
 
 
0.1
 
 
0.7
 
 
6.0
 
 
 
 
  Table S19.  Summary statistics (in mm) of the width
and thickness measurements recorded on bladelets.  SD  stands for
standard deviation. 
   
  
 Figure S10.  Boxplots showing the distribution of
platform width ( A ) and thickness ( B )
values across the studied sequence. The figure includes results of the
Kruskal-Wallis test and the pairwise comparisons. Statistically
significant differences are observed when comparing both  ars 
and  gic  to  rsa’ . 
   
 
 
 
 
 
 
Bulb type
 
 
 
 
 
 
 
 
 
 
 
 
Layer
 
 
Absent
 
 
Moderate
 
 
Pronounced
 
 
Total
 
 
 
 
ars
 
 
35 (81.4%)
 
 
7 (16.3%)
 
 
1 (2.3%)
 
 
43 (100.0%)
 
 
 
 
gic
 
 
355 (62.9%)
 
 
200 (35.5%)
 
 
9 (1.6%)
 
 
564 (100.0%)
 
 
 
 
rsa’
 
 
149 (53.8%)
 
 
123 (44.4%)
 
 
5 (1.8%)
 
 
277 (100.0%)
 
 
 
 
  Table S20.  Presence and type of bulbs recorded on
bladelets. A Pearson’s chi-squared test reveals significant differences
between layers (Chi-squared = 15.14,  p  = 0.004). 
   
 
 
 
 
 
 
Lip type
 
 
 
 
 
 
 
 
 
 
 
 
Layer
 
 
Absent
 
 
Moderate
 
 
Pronounced
 
 
Total
 
 
 
 
ars
 
 
2 (4.7%)
 
 
32 (74.4%)
 
 
9 (20.9%)
 
 
43 (100.0%)
 
 
 
 
gic
 
 
63 (11.2%)
 
 
419 (74.3%)
 
 
82 (14.5%)
 
 
564 (100.0%)
 
 
 
 
rsa’
 
 
48 (17.3%)
 
 
183 (66.1%)
 
 
46 (16.6%)
 
 
277 (100.0%)
 
 
 
 
  Table S21.  Presence and type of lips recorded on
bladelets. A Pearson’s chi-squared test reveals significant differences
between layers (Chi-squared = 11.02,  p  = 0.03). 
   
 
 
 Tables and figures reporting technological and morphological
attributes 
   
 
 
 
 
 
 
Scar pattern
 
 
 
 
 
 
 
 
 
 
 
 
 
 
Layer
 
 
Unidirectional parallel
 
 
Unidirectional convergent
 
 
Bidirectional
 
 
Other
 
 
Total
 
 
 
 
ars
 
 
23 (53.5%)
 
 
18 (41.9%)
 
 
0 (0.0%)
 
 
2 (4.7%)
 
 
43 (100.0%)
 
 
 
 
gic
 
 
237 (42.0%)
 
 
308 (54.6%)
 
 
5 (0.9%)
 
 
14 (2.5%)
 
 
564 (100.0%)
 
 
 
 
rsa’
 
 
138 (49.8%)
 
 
119 (43.0%)
 
 
4 (1.4%)
 
 
16 (5.8%)
 
 
277 (100.0%)
 
 
 
 
  Table S22.  Scar patterns recorded on the bladelet
assemblages. The  Other  category includes scar patterns found in
low frequencies (e.g., crossed, unidirectional transverse, and
undetermined patterns). The result of a Pearson’s chi-squared test
reveals significant differences between layers (Chi-squared = 15.61,
 p  = 0.02). 
   
 
 
 
 
 
 
Blank shape
 
 
 
 
 
 
 
 
 
 
 
 
 
 
Layer
 
 
Sub-parallel
 
 
Convergent
 
 
Irregular
 
 
Other
 
 
Total
 
 
 
 
ars
 
 
15 (39.5%)
 
 
12 (31.6%)
 
 
3 (7.9%)
 
 
8 (21.1%)
 
 
38 (100.0%)
 
 
 
 
gic
 
 
185 (40.8%)
 
 
160 (35.3%)
 
 
53 (11.7%)
 
 
55 (12.1%)
 
 
453 (100.0%)
 
 
 
 
rsa’
 
 
117 (46.4%)
 
 
81 (32.1%)
 
 
37 (14.7%)
 
 
17 (6.7%)
 
 
252 (100.0%)
 
 
 
 
  Table S23.  External shape recorded on blades. The
category  Other  includes categories found in low frequencies
(e.g., convex, comma-like). A Pearson’s chi-squared test reveals no
differences between layers (Chi-squared = 11.98,  p  = 0.06). 
   
 
 
 
 
 
 
Distal end
 
 
 
 
 
 
 
 
 
 
 
 
 
 
Layer
 
 
Convex
 
 
Irregular
 
 
Pointed
 
 
Straight
 
 
Total
 
 
 
 
ars
 
 
15 (39.5%)
 
 
1 (2.6%)
 
 
14 (36.8%)
 
 
8 (21.1%)
 
 
38 (100.0%)
 
 
 
 
gic
 
 
163 (36.0%)
 
 
39 (8.6%)
 
 
181 (40.0%)
 
 
70 (15.5%)
 
 
453 (100.0%)
 
 
 
 
rsa’
 
 
91 (36.1%)
 
 
16 (6.3%)
 
 
94 (37.3%)
 
 
51 (20.2%)
 
 
252 (100.0%)
 
 
 
 
  Table S24.  Distal end shape (in dorsal view)
recorded on bladelet. A Pearson’s chi-squared test reveals no
differences between layers (Chi-squared = 5.28,  p  = 0.51). 
   
 
 
 
 
 
 
Curvature
 
 
 
 
 
 
 
 
 
 
 
 
Layer
 
 
Curved
 
 
Curved slightly
 
 
Straight
 
 
Total
 
 
 
 
ars
 
 
18 (41.9%)
 
 
11 (25.6%)
 
 
14 (32.6%)
 
 
43 (100.0%)
 
 
 
 
gic
 
 
166 (29.4%)
 
 
166 (29.4%)
 
 
232 (41.1%)
 
 
564 (100.0%)
 
 
 
 
rsa’
 
 
111 (40.1%)
 
 
74 (26.7%)
 
 
92 (33.2%)
 
 
277 (100.0%)
 
 
 
 
  Table S25.  Presence and intensity of profile
curvature recorded on complete bladelet. The result of a Pearson’s
chi-squared test reveals significant differences between layers
(Chi-squared = 11.32,  p  = 0.02). 
   
 
 
 
 
 
 
Torsion simplified
 
 
 
 
 
 
 
 
 
 
Layer
 
 
no
 
 
yes
 
 
Total
 
 
 
 
ars
 
 
39 (90.7%)
 
 
4 (9.3%)
 
 
43 (100.0%)
 
 
 
 
gic
 
 
453 (80.3%)
 
 
111 (19.7%)
 
 
564 (100.0%)
 
 
 
 
rsa’
 
 
198 (71.5%)
 
 
79 (28.5%)
 
 
277 (100.0%)
 
 
 
 
  Table S26.  Presence of profile twisting recorded on
complete bladelets. The result of a Pearson’s chi-squared test reveals
significant differences between layers (Chi-squared = 12.69,  p 
= 0.002). 
   
 
 
 
 
 
 
Cross-section
 
 
 
 
 
 
 
 
 
 
 
 
 
 
 
 
Layer
 
 
Flat
 
 
Lateral steep
 
 
Polyhedral
 
 
Trapezoidal
 
 
Triangular
 
 
Total
 
 
 
 
ars
 
 
4 (9.3%)
 
 
5 (11.6%)
 
 
1 (2.3%)
 
 
18 (41.9%)
 
 
15 (34.9%)
 
 
43 (100.0%)
 
 
 
 
gic
 
 
22 (3.9%)
 
 
31 (5.5%)
 
 
28 (5.0%)
 
 
234 (41.5%)
 
 
249 (44.1%)
 
 
564 (100.0%)
 
 
 
 
rsa’
 
 
15 (5.4%)
 
 
37 (13.4%)
 
 
23 (8.3%)
 
 
129 (46.6%)
 
 
73 (26.4%)
 
 
277 (100.0%)
 
 
 
 
  Table S27.  Cross-section shape recorded on
bladelets. A Pearson’s chi-squared test reveals significant differences
between layers (Chi-squared = 38.74,  p  < 0.01). 
   
 
 
 
 
Layer
 
 
variable
 
 
n
 
 
mean
 
 
sd
 
 
min
 
 
median
 
 
max
 
 
 
 
 
 
ars
 
 
Elongation
 
 
38
 
 
2.2
 
 
0.7
 
 
1.2
 
 
2.1
 
 
4.4
 
 
 
 
ars
 
 
Robustness
 
 
38
 
 
4.0
 
 
1.4
 
 
1.3
 
 
4.0
 
 
7.7
 
 
 
 
gic
 
 
Elongation
 
 
453
 
 
2.4
 
 
0.8
 
 
1.1
 
 
2.2
 
 
8.7
 
 
 
 
gic
 
 
Robustness
 
 
453
 
 
3.7
 
 
1.1
 
 
1.0
 
 
3.7
 
 
8.3
 
 
 
 
rsa’
 
 
Elongation
 
 
252
 
 
2.9
 
 
0.9
 
 
1.1
 
 
2.7
 
 
6.9
 
 
 
 
rsa’
 
 
Robustness
 
 
252
 
 
3.5
 
 
1.3
 
 
0.8
 
 
3.4
 
 
7.4
 
 
 
 
  Table S28.  Summary statistics of the elongation
(length to width ratio) and robustness (width to thickness ratio) of
bladelets.  SD  stands for standard deviation. 
   
  
 Figure S11.  Boxplots showing the distribution of
elongation ( A ) and robustness ( B )
ratios across the studied sequence. The figure includes results of the
Kruskal-Wallis test and the pairwise comparisons. Statistically
significant differences are observed when comparing both  ars 
and  gic  to  rsa’ . 
   
 
 
 Tables and figures reporting metric attributes 
   
 
 
 
 
Layer
 
 
variable
 
 
n
 
 
mean
 
 
sd
 
 
min
 
 
median
 
 
max
 
 
 
 
 
 
ars
 
 
Length
 
 
38
 
 
14.0
 
 
5.7
 
 
5.5
 
 
13.0
 
 
30.5
 
 
 
 
ars
 
 
Width
 
 
38
 
 
6.5
 
 
2.4
 
 
2.8
 
 
6.2
 
 
11.3
 
 
 
 
ars
 
 
Thickness
 
 
38
 
 
1.8
 
 
0.9
 
 
0.7
 
 
1.7
 
 
5.1
 
 
 
 
gic
 
 
Length
 
 
453
 
 
15.5
 
 
5.6
 
 
6.3
 
 
14.5
 
 
38.2
 
 
 
 
gic
 
 
Width
 
 
453
 
 
6.7
 
 
2.0
 
 
2.1
 
 
6.6
 
 
12.0
 
 
 
 
gic
 
 
Thickness
 
 
453
 
 
1.9
 
 
0.8
 
 
0.6
 
 
1.7
 
 
6.2
 
 
 
 
rsa’
 
 
Length
 
 
252
 
 
20.9
 
 
7.7
 
 
6.9
 
 
19.4
 
 
44.2
 
 
 
 
rsa’
 
 
Width
 
 
252
 
 
7.3
 
 
2.2
 
 
1.9
 
 
7.4
 
 
11.9
 
 
 
 
rsa’
 
 
Thickness
 
 
252
 
 
2.4
 
 
1.4
 
 
0.7
 
 
2.1
 
 
9.1
 
 
 
 
  Table S29.  Summary statistics of linear dimensions
(length, width, and thickness in mm) recorded on complete bladelets,
excluding those modified by lateral retouch.  SD  stands for
standard deviation. 
   
  
 Figure S12.  Boxplots showing the distribution of length
( A ), width ( B ), and thickness
( C ) across the studied sequence. The figure includes
results of the Kruskal-Wallis test and the pairwise comparisons.
Statistically significant differences are observed when comparing both
 ars  and  gic  to  rsa’ . 
   
 
 
 
 
 Tools 
 
 Common tools 
   
  
 Figure S13.  Selection of tools and core-tools from
 rsa’ . The number following the alphabetical list corresponds to
the ID assigned by AF during the techno-typological analysis (refer to
the provided dataset for details). The figure includes: multiple burin
on prepared platform (a), busked burin (b), carinated endscraper (c),
endscrapers (d-e), blade with Aurignacian retouch (f), retouched blade
(g), and retouched flake (h). Drawings are from Gambassini (1997). 
   
  
 Figure S14.  Selection of tools and core-tools from
 gic  (a-e, g-j) and  ars  (f, i). The number following
the alphabetical list corresponds to the ID assigned by AF during the
techno-typological analysis (refer to the provided dataset for details).
The figure includes: endscrapers (a, c, e), thick-nosed endscrapers (b,
d), endscraper on a retouched flake (f), carinated endscraper (g),
retouched blade (h), retouched flake (i), and truncaton (j). Drawings
are from Gambassini (1997). 
   
  
 Figure S15.  Carinated burins recovered in  gic 
(a) and  ars  (b). The number following the alphabetical list
corresponds to the ID assigned by AF during the techno-typological
analysis (refer to the provided dataset for details). Drawings are from
Gambassini (1997). 
   
 
 
 
 
 
 
Blank
 
 
 
 
 
 
 
 
 
 
 
 
 
 
Layer
 
 
Blade
 
 
Flake
 
 
Other
 
 
Undetermined
 
 
Total
 
 
 
 
ars
 
 
1 (8.3%)
 
 
11 (91.7%)
 
 
0 (0.0%)
 
 
0 (0.0%)
 
 
12 (100.0%)
 
 
 
 
gic
 
 
29 (27.9%)
 
 
66 (63.5%)
 
 
4 (3.8%)
 
 
5 (4.8%)
 
 
104 (100.0%)
 
 
 
 
rsa’
 
 
34 (34.3%)
 
 
58 (58.6%)
 
 
3 (3.0%)
 
 
4 (4.0%)
 
 
99 (100.0%)
 
 
 
 
  Table S30.  Classification of common tools according
to the type of blank selected. The  Other  category includes
several carinated pieces made on pebbles and block fragments. Bladelets
are excluded from the table. 
   
 
 
 
 
 
 
Technology
 
 
 
 
 
 
 
 
 
 
 
 
 
 
 
 
 
 
Layer
 
 
Optimal
 
 
Initialization
 
 
Semi-cortical
 
 
Maintenance
 
 
Other
 
 
Undetermined
 
 
Total
 
 
 
 
ars
 
 
18 (75.0%)
 
 
0 (0.0%)
 
 
2 (8.3%)
 
 
2 (8.3%)
 
 
0 (0.0%)
 
 
2 (8.3%)
 
 
24 (100.0%)
 
 
 
 
gic
 
 
245 (82.8%)
 
 
3 (1.0%)
 
 
21 (7.1%)
 
 
19 (6.4%)
 
 
5 (1.7%)
 
 
3 (1.0%)
 
 
296 (100.0%)
 
 
 
 
rsa’
 
 
181 (79.4%)
 
 
4 (1.8%)
 
 
19 (8.3%)
 
 
17 (7.5%)
 
 
4 (1.8%)
 
 
3 (1.3%)
 
 
228 (100.0%)
 
 
 
 
  Table S31.  Classification of common tools according
to the technological classification of blanks. The  Other 
category includes several carinated pieces made on pebbles and block
fragments. Bladelets are excluded from the table. Rounded percentages
are provided in brackets. 
   
 
 
 Retouched bladelets 
 Bladelets were predominantly selected from blanks belonging to
optimal reduction sequences, and only four bladelets had cortical
remains (Tables S32–S33). Castelcivita has provided an exceptional
number of complete retouched bladelets, especially in layer  gic 
(Table S34), representing one of the highest proportions of complete
bladelets within the Aurignacian (Falcucci et al., 2018). This variation
is not related to selective recovery strategies during archaeological
excavations, nor to sorting biases. All lithics were systematically
sorted by Gambassini (1997) and then by one of us (AF) without applying
any size cut-off. Various scenarios may explain the high frequency of
complete bladelets, ranging from minimal trampling and post-depositional
reworking of the sequence to specific site-use strategies. However, one
of the most likely scenario is that the miniaturized size of bladelets
may have prevented them from breaking. This is supported by the fact
that most of the complete bladelets come from the uppermost layers, but
also by the more common occurrence of mesial fragments in  rsa’ .
In this regard, it is noteworthy that mesial fragments are more common
in  rsa’ , likely reflecting differences in size between
assemblages. Future research will address these open questions,
considering spatial and functional data. 
   
 
 
 
 
 
 
Technology
 
 
 
 
 
 
 
 
 
 
 
 
Layer
 
 
Optimal
 
 
Semi-cortical
 
 
Maintenance
 
 
Total
 
 
 
 
ars
 
 
11 (91.7%)
 
 
0 (0.0%)
 
 
1 (8.3%)
 
 
12 (100.0%)
 
 
 
 
gic
 
 
188 (97.9%)
 
 
1 (0.5%)
 
 
3 (1.6%)
 
 
192 (100.0%)
 
 
 
 
rsa’
 
 
125 (96.9%)
 
 
2 (1.6%)
 
 
2 (1.6%)
 
 
129 (100.0%)
 
 
 
 
  Table S32.  Technological classification of bladelets
selected for retouching. Rounded percentages are provided in
brackets. 
   
 
 
 
 
 
 
Cortex
 
 
 
 
 
 
 
 
 
 
 
 
Layer
 
 
0%
 
 
1-33%
 
 
33-66%
 
 
Total
 
 
 
 
ars
 
 
11 (91.7%)
 
 
1 (8.3%)
 
 
0 (0.0%)
 
 
12 (100.0%)
 
 
 
 
gic
 
 
191 (99.5%)
 
 
0 (0.0%)
 
 
1 (0.5%)
 
 
192 (100.0%)
 
 
 
 
rsa’
 
 
127 (98.4%)
 
 
1 (0.8%)
 
 
1 (0.8%)
 
 
129 (100.0%)
 
 
 
 
  Table S33.  Classification of retouched bladelets
according to cortex coverage. Rounded percentages are provided in
brackets. 
   
 
 
 
 
 
 
Preservation
 
 
 
 
 
 
 
 
 
 
 
 
 
 
Layer
 
 
Complete
 
 
Proximal
 
 
Mesial
 
 
Distal
 
 
Total
 
 
 
 
ars
 
 
5 (41.7%)
 
 
3 (25.0%)
 
 
1 (8.3%)
 
 
3 (25.0%)
 
 
12 (100.0%)
 
 
 
 
gic
 
 
111 (57.8%)
 
 
37 (19.3%)
 
 
10 (5.2%)
 
 
34 (17.7%)
 
 
192 (100.0%)
 
 
 
 
rsa’
 
 
25 (19.4%)
 
 
46 (35.7%)
 
 
35 (27.1%)
 
 
23 (17.8%)
 
 
129 (100.0%)
 
 
 
 
  Table S34.  Classification of retouched bladelets
according to the degree of fragmentation. Rounded percentages are
provided in brackets. A Pearson’s chi-squared test reveals significant
differences between layers (Chi-squared = 62.40,  p  <
0.01). 
   
 
 
 
 
 
 
Retouch position
 
 
 
 
 
 
 
 
 
 
 
 
Layer
 
 
Alternate
 
 
Direct
 
 
Inverse
 
 
Total
 
 
 
 
ars
 
 
0 (0.0%)
 
 
8 (66.7%)
 
 
4 (33.3%)
 
 
12 (100.0%)
 
 
 
 
gic
 
 
11 (5.8%)
 
 
173 (90.6%)
 
 
7 (3.7%)
 
 
191 (100.0%)
 
 
 
 
rsa’
 
 
26 (20.8%)
 
 
22 (17.6%)
 
 
77 (61.6%)
 
 
125 (100.0%)
 
 
 
 
  Table S35.  Position of retouch on bladelets across
the studied assemblages. Rounded percentages are provided in brackets. A
Pearson’s chi-squared test reveals significant differences between
layers (Chi-squared = 176.33,  p  < 0.01). 
   
 
 
 
 
Layer
 
 
Bilateral
 
 
Unilateral
 
 
Total
 
 
 
 
 
 
ars
 
 
4 (50.0%)
 
 
4 (50.0%)
 
 
8 (100.0%)
 
 
 
 
gic
 
 
109 (63.0%)
 
 
64 (37.0%)
 
 
173 (100.0%)
 
 
 
 
rsa’
 
 
10 (45.5%)
 
 
12 (54.5%)
 
 
22 (100.0%)
 
 
 
 
  Table S36.  Position of the direct retouch across the
modified bladelets. Rounded percentages are provided in brackets. 
   
  
 Figure S16.  Selection of retouched bladelets from
 gic . The number following the alphabetical list corresponds to
the ID assigned by A. Falcucci during the techno-typological analysis
(refer to the provided Dataset for details). Tools have been sorted by
retouch position: direct bilateral retouch (a-d, h-i, m, p-z, and aa),
direct unilateral (e-g, l, and o), inverse (j), and alternate (k and n).
Photos by A. Falcucci. 
   
  
 Figure S17.  Selection of retouched bladelets from
 ars . The number following the alphabetical list corresponds to
the ID assigned by A.Falcucci during the techno-typological analysis
(refer to the provided dataset for details). Tools have been sorted by
retouch position: direct bilateral retouch (a), direct unilateral (b),
and inverse (c). Photos by A. Falcucci. 
   
 
 
 
 
Layer
 
 
variable
 
 
n
 
 
mean
 
 
sd
 
 
min
 
 
median
 
 
max
 
 
 
 
 
 
ars
 
 
Length
 
 
5
 
 
19.7
 
 
15.2
 
 
9.3
 
 
13.3
 
 
46.1
 
 
 
 
gic
 
 
Length
 
 
111
 
 
13.2
 
 
5.0
 
 
5.0
 
 
11.7
 
 
34.7
 
 
 
 
rsa’
 
 
Length
 
 
25
 
 
19.9
 
 
7.2
 
 
6.0
 
 
20.0
 
 
37.5
 
 
 
 
  Table S37.  Summary statistics of the length values
(in mm) recorded on complete retouched bladelets.  SD  stands for
standard deviation. 
   
 
 
 
 
Layer
 
 
variable
 
 
n
 
 
mean
 
 
sd
 
 
min
 
 
median
 
 
max
 
 
 
 
 
 
ars
 
 
Width
 
 
12
 
 
6.4
 
 
2.6
 
 
4.0
 
 
5.0
 
 
11.5
 
 
 
 
ars
 
 
Thickness
 
 
12
 
 
2.0
 
 
1.1
 
 
0.8
 
 
1.8
 
 
4.3
 
 
 
 
gic
 
 
Width
 
 
192
 
 
5.1
 
 
1.6
 
 
2.2
 
 
4.8
 
 
11.1
 
 
 
 
gic
 
 
Thickness
 
 
192
 
 
1.6
 
 
0.6
 
 
0.5
 
 
1.5
 
 
5.3
 
 
 
 
rsa’
 
 
Width
 
 
129
 
 
6.5
 
 
2.0
 
 
2.7
 
 
6.4
 
 
11.5
 
 
 
 
rsa’
 
 
Thickness
 
 
129
 
 
1.8
 
 
0.7
 
 
0.5
 
 
1.7
 
 
4.1
 
 
 
 
  Table S38.  Summary statistics of linear dimensions
(width, and thickness in mm) recorded on retouched bladelets.
 SD  stands for standard deviation. 
   
  
 Figure S18.  Boxplots showing the distribution of width
( A ) and thickness ( B ) values of
retouched bladelets across the studied sequence. The figure includes
results of the Kruskal-Wallis test and pairwise comparisons.
Statistically significant differences are observed when comparing
 gic  to  rsa’ . However, it’s important to note that the
number of available retouched bladelets in  ars  is very small,
making it challenging to draw meaningful conclusions concerning the
obtained pairwise comparisons. 
   
 
 
 
 2DGM analysis 
   
  
 Figure S19.  Scree plot showing the proportion of
variance explained by the first four principal components. 
   
  
 Figure S20.  Scatterplot showing the correlation
(Spearman test) between the length and PC1 across the studied assemblage
of complete bladelets. 
   
 
 Mean shapes 
 Studying the mean shapes is important to highlight the association
between the modified and unmodified bladelets recovered in the studied
layers. The comparison between unmodified bladelets and bladelets with
direct retouch from gic is rather straightforward and illustrates the
knappers’ interest in obtaining a convergent distal edge. On the other
hand, bladelets with direct retouch from rsa’ appear to be wider,
especially on the proximal side, compared to the mean shape of
unretouched blanks from the same layer. These findings are further
supported by the principal component analysis (PCA) visualized in the
paper. The variability identified along PC1 in the bivariate plot in
Fig. S18 highlights the effects of retouching on the overall shape of
artifacts, as well as the close relation between blanks from gic and all
bladelets modified by direct bilateral retouch. 
    
 Figure S21.  Confusion matrix displaying comparisons
between mean shapes of bladelet groups analyzed using 2DGM. The gray
shapes in the diagonal represent the mean shapes for each group, while
the comparisons are colored yellow (along the x axis) and red (along the
y axis). The asterisk symbol marks statistically significant comparisons
based on pairwise tests conducted following the PERMANOVA test. The plot
was first generated in R using the  Momocs  package (Bonhomme et
al., 2014) and then redrawn in Adobe Illustrator to improve the quality
and readability. The raw PDF file is available in the associated
research compendium. 
   
 
 
 
 
 References 
 Andersen K.K., Svensson A., Johnsen S.J., Rasmussen S.O., Bigler M.,
Röthlisberger R., Ruth U., Siggaard-Andersen M.-L., Peder Steffensen J.,
Dahl-Jensen D., Vinther B.M. & Clausen H.B. (2006) The Greenland Ice
Core Chronology 2005, 15–42ka. Part 1: constructing the time scale.
Quaternary Science Reviews, 25: 3246-3257.  doi:https://doi.org/10.1016/j.quascirev.2006.08.002  
 Arrighi S., Marciani G., Rossini M., Pereira Santos M.C., Fiorini A.,
Martini I., Aureli D., Badino F., Bortolini E., Figus C., Lugli F.,
Oxilia G., Romandini M., Silvestrini S., Ronchitelli A., Moroni A. &
Benazzi S. (2020) Between the hammerstone and the anvil: bipolar
knapping and other percussive activities in the late Mousterian and the
Uluzzian of Grotta di Castelcivita (Italy). Archaeological and
Anthropological Sciences, 12: 271.  doi:10.1007/s12520-020-01216-w  
 Badino F., Pini R., Ravazzi C., Margaritora D., Arrighi S., Bortolini
E., Figus C., Giaccio B., Lugli F., Marciani G., Monegato G., Moroni A.,
Negrino F., Oxilia G., Peresani M., Romandini M., Ronchitelli A.,
Spinapolice E.E., Zerboni A. & Benazzi S. (2020) An overview of
Alpine and Mediterranean palaeogeography, terrestrial ecosystems and
climate history during MIS 3 with focus on the Middle to Upper
Palaeolithic transition. Quaternary International, 551: 7-28.  doi:https://doi.org/10.1016/j.quaint.2019.09.024  
 Bon F. & Bodu P. (2002) Analyse technologique du débitage
aurignacien. In B. Schmider (ed.): L’Aurignacien de la grotte du Renne.
Les fouilles d’André Leroi-Gourhan à Arcy-sur-Cure (Yonne), pp. 115-133.
CNRS, Paris. 
 Bonhomme V., Picq S., Gaucherel C. & Claude J. (2014) Momocs:
Outline analysis using R. Journal of Statistical Software, 56: 1-24.  doi:https://doi.org/10.18637/jss.v056.i13  
 Cafaro S., Gueguen E., Parise M. & Schiattarella M. (2016)
Morphometric analysis of Karst 98 features of the Alburni Mts, Southern
Apennines, Italy. Geografia Fisica e Dinamica Quaternaria, 39: 121-128.
 doi:https://doi.org/10.4461/GFDQ2016.39.11  
 d’Errico F. & Banks W.E. (2015) Tephra studies and the
reconstruction of Middle-to-Upper Paleolithic cultural trajectories.
Quaternary Science Reviews, 118: 182-193.  doi:10.1016/j.quascirev.2014.05.014  
 Discamps E., Jaubert J. & Bachellerie F. (2011) Human choices and
environmental constraints: deciphering the variability of large game
procurement: from Mousterian to Aurignacian times (MIS 5-3) in
southwestern France. Quaternary Science Reviews, 30: 2755-2775.  doi:10.1016/j.quascirev.2011.06.009  
 Douka K., Higham T., Wood R., Boscato P., Gambassini P., Karkanas P.,
Peresani M. & Ronchitelli A.M. (2014) On the chronology of the
Uluzzian. Journal Of Human Evolution, 68: 1-13.  doi:10.1016/j.jhevol.2013.12.007  
 Falcucci A., Conard N.J. & Peresani M. (2017) A critical
assessment of the Protoaurignacian lithic technology at Fumane Cave and
its implications for the definition of the earliest Aurignacian. PLoS
One, 12: e0189241.  doi:10.1371/journal.pone.0189241  
 Falcucci A., Peresani M., Roussel M., Normand C. & Soressi M.
(2018) What’s the point? Retouched bladelet variability in the
Protoaurignacian. Results from Fumane, Isturitz, and Les Cottés.
Archaeological and Anthropological Sciences, 10: 539-554.  doi:10.1007/s12520-016-0365-5  
 Fedele F., Giaccio B., Isaia R. & Orsi G. (2004) The Campanian
Ignimbrite Eruption, Heinrich Event 4, and Palaeolithic Change in
Europe: A High-Resolution Investigation. Volcanism and the Earth’s
Atmosphere, 139: 301-325.  doi:doi:10.1029/139GM20  
 Fiore I., Gala M., Boschin F., Crezzini J., Tagliacozzo A. &
Moroni A. (2020) Archeozoology and taphonomy of bird remains from Grotta
di Castelcivita (Salerno, Italy) and clues for human-bird interactions.
Quaternary International, 551: 224-242.  doi:https://doi.org/10.1016/j.quaint.2019.09.004  
 Gambassini P. (1997) Il Paleolitico di Castelcivita: Culture e
Ambiente. Electa, Naples 
 Giaccio B., Isaia R., Fedele F.G., Di Canzio E., Hoffecker J.,
Ronchitelli A., Sinitsyn A.A., Anikovich M., Lisitsyn S.N. & Popov
V.V. (2008) The Campanian Ignimbrite and Codola tephra layers: Two
temporal/stratigraphic markers for the Early Upper Palaeolithic in
southern Italy and eastern Europe. Journal of Volcanology and Geothermal
Research, 177: 208-226.  doi:https://doi.org/10.1016/j.jvolgeores.2007.10.007  
 Giaccio B., Hajdas I., Isaia R., Deino A. & Nomade S. (2017)
High-precision 14C and 40Ar/39Ar dating of the Campanian Ignimbrite
(Y-5) reconciles the time-scales of climatic-cultural processes at 40
ka. Scientific Reports, 7: 45940.  doi:10.1038/srep45940  
 Kolobova K.A., Krivoshapkin A.I. & Pavlenok K.K. (2014) Carinated
Pieces in Paleolithic Assemblages of Central Asia1. Archaeology,
Ethnology and Anthropology of Eurasia, 42: 13-29.  doi:10.1016/j.aeae.2015.06.003  
 Le Brun-Ricalens F. (2005) Chronique d’une reconnaissance attendue.
Outils “carénés”, outils “nucléiformes”: nucléus à lamelles. Bilan après
un siècle de recherches typologiques, technologiques et tracéologiques.
In F. Le Brun-Ricalens (ed.): Productions lamellaires attribuées à
l’Aurignacien. MNHA, Luxembourg. 
 López-García J.M., dalla Valle C., Cremaschi M. & Peresani M.
(2015) Reconstruction of the Neanderthal and Modern Human landscape and
climate from the Fumane cave sequence (Verona, Italy) using small-mammal
assemblages. Quaternary Science Reviews, 128: 1-13.  doi:http://doi.org/10.1016/j.quascirev.2015.09.013  
 Lowe J., Barton N., Blockley S., Ramsey C.B., Cullen V.L., Davies W.,
Gamble C., Grant K., Hardiman M., Housley R., Lane C.S., Lee S., Lewis
M., MacLeod A., Menzies M., Müller W., Pollard M., Price C., Roberts
A.P., Rohling E.J., Satow C., Smith V.C., Stringer C.B., Tomlinson E.L.,
White D., Albert P., Arienzo I., Barker G., Borić D., Carandente A.,
Civetta L., Ferrier C., Guadelli J.-L., Karkanas P., Koumouzelis M.,
Müller U.C., Orsi G., Pross J., Rosi M., Shalamanov-Korobar L., Sirakov
N. & Tzedakis P.C. (2012) Volcanic ash layers illuminate the
resilience of Neanderthals and early modern humans to natural hazards.
Proceedings of the National Academy of Sciences, 109: 13532-13537.  doi:10.1073/pnas.1204579109  
 Margari V., Gibbard P.L., Bryant C.L. & Tzedakis P.C. (2009)
Character of vegetational and environmental changes in southern Europe
during the last glacial period; evidence from Lesvos Island, Greece.
Quaternary Science Reviews, 28: 1317-1339.  doi:https://doi.org/10.1016/j.quascirev.2009.01.008  
 Marín-Arroyo A.B., Terlato G., Vidal-Cordasco M. & Peresani M.
(2023) Subsistence of early anatomically modern humans in Europe as
evidenced in the Protoaurignacian occupations of Fumane Cave, Italy.
Scientific Reports, 13: 3788.  doi:10.1038/s41598-023-30059-3  
 Martini I., Ronchitelli A., Arrighi S., Capecchi G., Ricci S.,
Scaramucci S., Spagnolo V., Gambassini P. & Moroni A. (2018) Cave
clastic sediments as a tool for refining the study of human occupation
of prehistoric sites: insights from the cave site of La Cala (Cilento,
southern Italy). Journal of Quaternary Science, 33: 586-596.  doi:10.1002/jqs.3038  
 Martini I., Baucon A. & Boschin F. (2021) Depositional processes
and environmental settings in rock shelters: the case of the prehistoric
Oscurusciuto site (Southern Italy). Geological Magazine, 158: 891-904.
 doi:10.1017/s0016756820001041  
 Palma di Cesnola A. (2004) Paglicci: l’Aurignaziano e il Gravettiano
antico. Claudio Grenzi, Foggia 
 Peresani M., Bertola S., Delpiano D., Benazzi S. & Romandini M.
(2019) The Uluzzian in the north of Italy: insights around the new
evidence at Riparo Broion. Archaeological and Anthropological Sciences,
11: 3503-3536.  doi:10.1007/s12520-018-0770-z  
 Rasmussen S.O., Bigler M., Blockley S.P., Blunier T., Buchardt S.L.,
Clausen H.B., Cvijanovic I., Dahl-Jensen D., Johnsen S.J., Fischer H.,
Gkinis V., Guillevic M., Hoek W.Z., Lowe J.J., Pedro J.B., Popp T.,
Seierstad I.K., Steffensen J.P., Svensson A.M., Vallelonga P., Vinther
B.M., Walker M.J.C., Wheatley J.J. & Winstrup M. (2014) A
stratigraphic framework for abrupt climatic changes during the Last
Glacial period based on three synchronized Greenland ice-core records:
refining and extending the INTIMATE event stratigraphy. Quaternary
Science Reviews, 106: 14-28.  doi:https://doi.org/10.1016/j.quascirev.2014.09.007  
 Romandini M., Crezzini J., Bortolini E., Boscato P., Boschin F.,
Carrera L., Nannini N., Tagliacozzo A., Terlato G., Arrighi S., Badino
F., Figus C., Lugli F., Marciani G., Oxilia G., Moroni A., Negrino F.,
Peresani M., Riel-Salvatore J., Ronchitelli A., Spinapolice E.E. &
Benazzi S. (2020) Macromammal and bird assemblages across the late
Middle to Upper Palaeolithic transition in Italy: an extended
zooarchaeological review. Quaternary International, 551: 188-223.  doi:https://doi.org/10.1016/j.quaint.2019.11.008  
 Rossini M., Marciani G., Arrighi S., Pereira Santos M.C., Spagnolo
V., Ronchitelli A., Benazzi S. & Moroni A. (2022) Less is more!
Uluzzian technical behaviour at the cave site of Castelcivita (southern
Italy). Journal of Archaeological Science: Reports, 44: 103494.  doi:https://doi.org/10.1016/j.jasrep.2022.103494  
 Skinner L.C. & Elderfield H. (2007) Rapid fluctuations in the
deep North Atlantic heat budget during the last glacial period.
Paleoceanography, 22.  doi:https://doi.org/10.1029/2006PA001338  
 Sparks S., Self S., Grattan J., Oppenheimer C., Pyle D. & Rymer
H. (2005) Super-eruptions: global effects and future threats: Report of
a Geological Society of London Working Group., 2nd edn., 
 Svensson A., Andersen K.K., Bigler M., Clausen H.B., Dahl-Jensen D.,
Davies S.M., Johnsen S.J., Muscheler R., Parrenin F., Rasmussen S.O.,
Röthlisberger R., Seierstad I., Steffensen J.P. & Vinther B.M.
(2008) A 60 000 year Greenland stratigraphic ice core chronology. Clim.
Past, 4: 47-57.  doi:10.5194/cp-4-47-2008  
 Wood R.E., Douka K., Boscato P., Haesaerts P., Sinitsyn A. &
Higham T. (2012) Testing the ABOx-SC method: Dating known-age charcoals
associated with the Campanian Ignimbrite. Quaternary Geochronology, 9:
16-26.  doi:https://doi.org/10.1016/j.quageo.2012.02.003  
 Wulf S., Hardiman M.J., Staff R.A., Koutsodendris A., Appelt O.,
Blockley S.P.E., Lowe J.J., Manning C.J., Ottolini L., Schmitt A.K.,
Smith V.C., Tomlinson E.L., Vakhrameeva P., Knipping M., Kotthoff U.,
Milner A.M., Müller U.C., Christanis K., Kalaitzidis S., Tzedakis P.C.,
Schmiedl G. & Pross J. (2018) The marine isotope stage 1–5
cryptotephra record of Tenaghi Philippon, Greece: Towards a detailed
tephrostratigraphic framework for the Eastern Mediterranean region.
Quaternary Science Reviews, 186: 236-262.  doi:https://doi.org/10.1016/j.quascirev.2018.03.011  
 Wutke K., Wulf S., Tomlinson E.L., Hardiman M., Dulski P.,
Luterbacher J. & Brauer A. (2015) Geochemical properties and
environmental impacts of seven Campanian tephra layers deposited between
40 and 38 ka BP in the varved lake sediments of Lago Grande di
Monticchio, southern Italy. Quaternary Science Reviews, 118: 67-83.  doi:https://doi.org/10.1016/j.quascirev.2014.05.017  
 


 
 

 

 

 

 

 

 

 
 

 
 
